# Microbial characteristics of bile in gallstone patients: a comprehensive analysis of 9,939 cases

**DOI:** 10.3389/fmicb.2024.1481112

**Published:** 2024-12-19

**Authors:** Xin Zheng, Yunjun Yan, Xin Li, Mimin Liu, Xiaoyue Zhao, Jing He, Xuewei Zhuang

**Affiliations:** ^1^Department of Clinical Laboratory, Shandong Provincial Third Hospital, Shandong University, Jinan, Shandong, China; ^2^Jinan Dian Medical Laboratory CO., LTD, Jinan, China; ^3^Second Clinical Medical College, Shandong University of Traditional Chinese Medicine, Jinan, Shandong, China; ^4^Jinan Key Laboratory for Precision Medicine, Jinan, Shandong, China

**Keywords:** gallstone, bile, microbes, clinical characteristics, recurrence

## Abstract

**Introduction:**

The exact triggers of gallstone formation remain incompletely understood, but research indicates that microbial infection is a significant factor and can interfere with treatment. There is no consensus on the bile microbial culture profiles in previous studies, and determining the microbial profile could aid in targeted prevention and treatment. The primary aim of this study is to investigate the differences in microbial communities cultured from bile specimens of patients with gallstones.

**Methods:**

We collected the clinical characteristics and bile microbial status of 9,939 gallstone patients. Statistical analysis was employed to assess the relationship between microbes and clinical features, and a random forest model was utilized to predict recurrence.

**Results:**

Results showed a higher proportion of females among patients, with the age group of 60-74 years being the most prevalent. The most common type of gallstone was solitary gallbladder stones. A total of 76 microbes were cultured from 5,153 patients, with *Escherichia coli, Klebsiella pneumoniae*, and *Enterococcus faecalis* being the most frequently identified. Significant differences in microbial diversity and positive detection rates were observed across different age groups, types of gallstones, and recurrence status. Positive frequencies of *E. coli, Enterococcus faecium*, and *K. pneumoniae* varied significantly by age group and gallstone type. The microbial diversity in the recurrence group was significantly lower compared to the non-recurrence group. The recurrence rate was significantly higher in the group with single microbial species compared to those with no microbes or multiple microbes. For the recurrence group, there were significant differences in the frequencies of seven microbes (*Aeromonas hydrophila, Enterococcus casseliflavus, Enterococcus faecium, E. coli, K. pneumoniae, Proteus mirabilis, Pseudomonas aeruginosa*) before and after recurrence, with these microbes appearing in a higher number of patients after recurrence. Regression analysis identified patient age, stone size, diabetes, venous thrombosis, liver cirrhosis, malignancy, coronary heart disease, and the number of microbial species as important predictors of recurrence. A random forest model constructed using these variables demonstrated good performance and high predictive ability (ROC-AUC = 0.862).

**Discussion:**

These findings highlight the significant role of microbial communities in gallstone formation and recurrence. Furthermore, the identified predictors of recurrence, including clinical factors and microbial diversity, may help develop personalized prevention and recurrence strategies for gallstone patients.

## 1 Introduction

Bile is a biological fluid primarily composed of bile acids (BA), cholesterol, phospholipids, and proteins. It is synthesized in the liver and stored in the gallbladder. Its main physiological function is to facilitate the absorption of fats in the small intestine during digestion (Begley et al., 2005). Several bile-related diseases can alter bile function, but the most common is the formation of gallstones in the gallbladder or bile ducts, known as cholelithiasis or Gallstone (GS) disease. GS disease is one of the most common biliary tract diseases and a major public health concern in many countries. The incidence rate of gallstones is rising because of the change of living standard. Previous ultrasonography (US)-based epidemiologic studies have shown prevalence rates of 9.7–19.5% in European countries, 10–12% in the United States, and 2–5% in Asian countries (Yoo and Lee, 2009; Ryu et al., 2016; Sacks et al., 2018). Nearly 75% of patients with gallstones have no obvious symptoms in the initial stages (Sun et al., 2022). According to the site of lithiasis, cholelithiasis is mainly divided into cholecystolithiasis, hepatolithiasis, and extrahepatic bile duct stones. As the gallstones progress in development, they may trigger symptoms such as nausea, epigastric colic, diarrhea, anorexia, etc. Eventually, gallstone obstruction can lead to life-threatening conditions such as acute cholangitis, acute cholecystitis, and biliary pancreatitis (Tanaka et al., 2018; De Simone et al., 2022). Until now many strategies, including traditional open surgery, laparoscopic operation, and robotic surgery, have been applied for the treatment of cholecystolithiasis with choledocholithiasis (Lv et al., 2016). However, each strategy has its advantages and disadvantages and none of the methods could provide a satisfactory effect. For instance, while cholecystectomy is commonly performed, it carries risks of complications that can significantly compromise a patient's health and overall quality of life (Shabanzadeh et al., 2016; Barahona Ponce et al., 2021).

The recurrence of gallstones is of paramount importance, with high recurrence rates and a wide range of recurrence times. According to studies, the recurrence rate after gallstone surgery is approximately 10% to 50%, and recurrence may occur within months to years after surgery (Boerma et al., 2002; Allen et al., 2006; Ye et al., 2020). Various factors may influence gallstone recurrence, including dietary habits, lifestyle, genetic factors, gallbladder dysfunction, obesity, changes in bile biochemical components, etc. (Cheon and Lehman, 2006). Gallstone recurrence has significant implications for patient health and medical management. Recurrence may lead to patients experiencing pain, discomfort, and complications again, and may even require repeat surgical treatment. Additionally, recurrence increases medical costs and treatment burden for patients, causing inconvenience and anxiety in their lives.

Although the exact mechanisms underlying the formation of gallstones remain incompletely understood, the process often involves multiple factors, including genetic predisposition, age, gender, excessive hepatic cholesterol secretion, impaired gallbladder motility, abnormal bile composition (Nardone et al., 1995; Wang et al., 2010; Shabanzadeh et al., 2017; Idowu et al., 2019; Granel-Villach et al., 2020). Patients with gallstones often have comorbidities such as diabetes and cardiovascular diseases. These conditions, due to metabolic abnormalities, inflammatory responses, impaired gallbladder motility, and the effects of medications, increase the likelihood of gallstone formation and elevate the risk of recurrence (Man et al., 2022; Meng and Liu, 2023; Portincasa et al., 2023; Zhu et al., 2023). In addition, microbes are increasingly recognized as a potential causative agent in the development of gallstone disease. In recent years, an increasing number of studies have found that microbes play a crucial role in the formation and progression of gallstones (Grigor'eva and Romanova, 2020). Studies have shown the presence of living bacteria in gallstones. The flora in the biliary tract and duodenum are highly homologous and closely related to the formation of gallstones. Microbes can enter the biliary system from the duodenum by migrating through the sphincter of Oddi. They can also spread hematogenously to the liver and from there into bile (Neri et al., 2005; Helaly et al., 2014). When in bile, microbes play an important role as nucleating factors, participating in the alteration of bile chemical composition and the nucleation of gallstones, which may lead to complications such as gallbladder or bile duct infections (Maurer et al., 2005).

The number of molecular studies focusing on the association between the biliary microbiome and gallstones has been increasing (Swidsinski and Lee, 2001; Stewart et al., 2002; Begley et al., 2005; Stewart et al., 2006; Wang et al., 2018; Shen et al., 2020). For example, a study by Liang et al. demonstrated that patients with Sphincter of Oddi laxity (SOL) had a more severe bacterial infection in the bile duct microenvironment and a higher lithogenic potential (Liang et al., 2016). The microbiota in the intestine, bile ducts, and gallbladder may contribute to gallstone formation (Shen et al., 2015; Kose et al., 2018; Molinero et al., 2019; Hu et al., 2022). Additionally, dysbiosis of the biliary microbiota has been associated with the recurrence of bile duct stones (Choe et al., 2021; Tan et al., 2022). Several studies have indicated that different bacterial species and bile compositions may increase the formation of recurrent common bile duct (CBD) stones (Swidsinski and Lee, 2001; Stewart et al., 2006).

Previous research has employed various techniques to study bile microbiota, such as microbial culture, polymerase chain reaction (PCR) targeting specific bacteria, 16S rRNA sequencing, metagenomics, and transmission electron microscopy. Conventional techniques like these have long been used to identify biliary microbiota, but most have focused on bacteria (Brook, 1989; Swidsinski and Lee, 2001; Stewart et al., 2002). As early as 1989, Brook et al. used culture methods to grow bacteria from 123 bile specimens, with the main bacteria being *Escherichia coli, group D Streptococcus, Klebsiella sp., Clostridium sp., Bacteroides sp*., and *Enterobacter sp*. (Brook, 1989). Wu et al. (2013) were the first to apply 16S rRNA sequencing to bile and gallstone samples from cholesterol gallstone patients. Another study reported unbiased metagenomic sequencing of bile samples from 15 patients with common bile duct stones, identifying 13 novel bile bacteria (Shen et al., 2015). A recent study using 16S rDNA sequencing found potentially harmful microbes (*Streptococcus, Clostridium*, and *Kocuria*) in gallstones collected during surgery that may cause postoperative complications (Ploszaj et al., 2021).

In recent years, research on the microbiota in gallstone bile has become increasingly abundant. Most studies are based on 16S rRNA, and using this technology, the bile bacterial profiles of healthy individuals and gallstone patients have been revealed, showing significant differences in the relative abundance of different groups between the two sample sets. In bile samples from control group patients, sequences belonging to the family Propionibacteriaceae were more abundant, while in bile samples from gallstone patients, sequences belonging to *Bacteroidaceae, Prevotellaceae, Porphyromonadaceae*, and *Veillonellaceae* were detected at higher frequencies (Molinero et al., 2019). The taxonomic composition of bile bacterial communities also showed significant differences between common bile duct stones and gallbladder stones (Park and Park, 2024). Moreover, a recent culture-based study found differences in bile microbiota between gallstone patients with and without complications (Hirata et al., 2023). Additionally, a recent study combining 16S rRNA gene sequencing and proteomics identified 158 microbial taxa in bile samples, discovering taxa such as *Streptococcus, Staphylococcus*, and *Clostridium*, which may contribute to gallstone formation, as well as bacteria involved in biofilm formation, such as *Helicobacter pylori, Cyanobacteria, Pseudomonas, E. coli*, and *Clostridium* (Yang et al., 2023).

To date, there have been no large cohorts characterizing the microbiota in gallstone bile, and the understanding of microbial differences among different clinical characteristics remains insufficient. This study uses clinical bile microbiology culture results from 9,939 gallstone patients to explore the bile microbiota characteristics in gallstone patients and compare microbial differences among different clinical characteristics. This may provide a deeper understanding of the role of microbiota in gallstones and their association with various clinical features, potentially offering more effective strategies for the prevention, diagnosis, and treatment of gallstones, thereby reducing patient suffering and medical burdens.

## 2 Methods

### 2.1 Study design and participants

**Figure 1:**
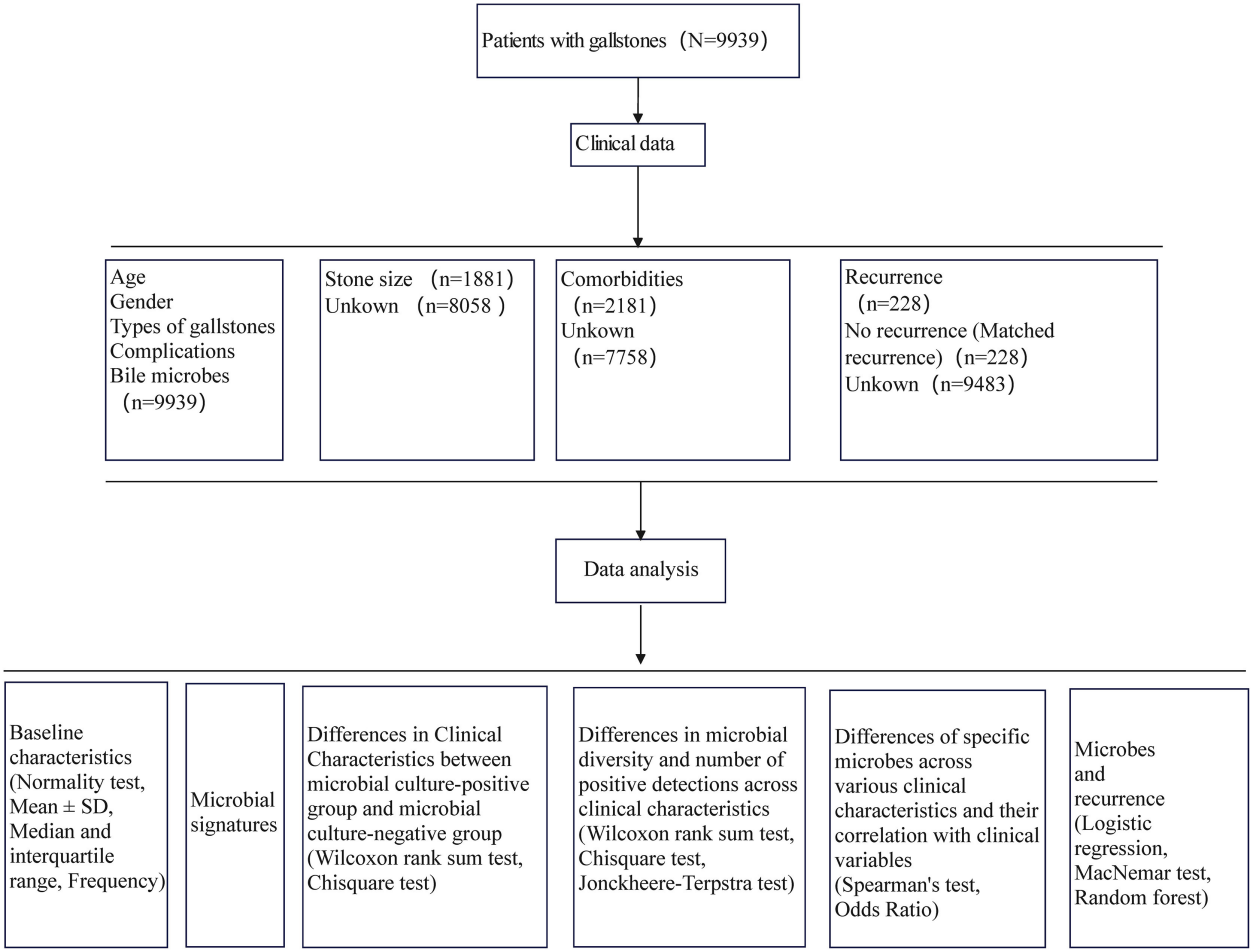
Study design.

A total of 9,939 patients diagnosed with gallstones and admitted for surgery between January 2017 and December 2023 at the Third Hospital of Shandong Province were included in this study. The study was approved by the Institutional Ethics Committee of the Third Hospital of Shandong Province (KYLL-2024064). Clinical data, including age, gender, type of gallstone, gallstone size, comorbidities, and bile culture microbiota information, were extracted from medical records (Supplementary Tables S1, S2). The study design is outlined in Figure 1.

### 2.2 Clinical definitions

#### 2.2.1 Gallstone type

Gallstones were classified based on their location into extrahepatic bile duct stones (EBD), hepatolithiasis bile duct stones (HBD), and cholecystolithiasis.

#### 2.2.2 Complications

In this study, complications refer to cholecystitis, cholangitis, and pancreatitis in gallstone patients.

#### 2.2.3 Recurrence

To avoid the impact of postoperative residual stones on recurrence, recurrence was defined as re-hospitalization and re-diagnosis of gallstones confirmed by imaging with no residual stones and more than 6 months after the initial surgery (von Schönfels et al., 2013). Recurrence patients were matched by gender and age with non-recurrence patients who had not been re-hospitalized for more than 2 years after surgery and were confirmed to have no recurrence through follow-up. All hospital admissions within 30 days were considered the same admission, while entries exceeding 30 days were defined as re-admissions (Shabanzadeh et al., 2019).

#### 2.2.4 Comorbidities

Comorbidities included hypertension, respiratory diseases, coronary heart disease, diabetes, renal cyst, cerebral infarction, fatty liver, venous thrombosis, gallbladder adenomyomatosis, gallbladder polyp, malignant tumor, and cirrhosis of the liver. The selection criterion was based on the top 12 most frequent comorbidities.

#### 2.2.5 Respiratory diseases

In this study, respiratory diseases included bronchitis, bronchiectasis, bronchial cyst, bronchial fistula, bronchial asthma, bronchial obstruction, solitary pulmonary nodule, emphysema, pneumonia, tuberculosis, atelectasis, pulmonary hypertension, chronic obstructive pulmonary disease, pulmonary fibrosis, pulmonary bullae, hamartoma, pulmonary embolism, interstitial lung disease, hilar lymphadenopathy, pulmonary edema, lung consolidation, polycystic lung, and pulmonary cavity.

#### 2.2.6 Malignant tumors

Malignant tumors in this study included thyroid malignancies, esophageal malignancies, gastric cardia malignancies, lung malignancies, breast malignancies, gastric malignancies, secondary peritoneal malignancies, secondary abdominal wall skin malignancies, secondary abdominal cavity malignancies, secondary abdominal lymph node malignancies, secondary mediastinal malignancies, secondary spinal malignancies, endometrial malignancies, liver malignancies, bile duct malignancies, gallbladder malignancies, bladder malignancies, pancreatic malignancies, colon malignancies, rectal malignancies, small intestine malignancies, duodenal malignancies, kidney malignancies, ureteral malignancies, secondary lymph node malignancies, secondary bone malignancies, prostate malignancies, cervical malignancies, and ovarian malignancies.

#### 2.2.7 Gallstone size

Gallstone size information was obtained for 1,891 participants, retaining only the longest diameter of the largest stone to represent stone size.

### 2.3 Microbial cultures

Bile samples were inoculated onto Columbia blood agar and MacConkey agar under sterile conditions for isolation and culture. Pathogens were purified and identified using an automated microbiology analyzer (VITEK2-COMPACT, BioMerieux, France). Quality control strains included *Klebsiella oxytoca* ATCC 700324 and *Enterococcus casseliflavus* ATCC 700327.

### 2.4 Statistical analysis

In descriptive statistics, normally distributed continuous variables are presented as mean ± SD, while non-normally distributed continuous variables are reported as median with interquartile range (IQR). Categorical variables are reported as percentages. Comparison of non-normally distributed continuous variables between two categorical variable groups was conducted using the Wilcoxon rank sum test. For comparison of categorical variables between two categorical variable groups, the Chi-square test was utilized. Analysis of differences in categorical variables among ordered categorical variable groups was performed using the Chi-square test, followed by *post-hoc* multiple comparisons if significant, and trends were analyzed using the Cochran-Armitage Trend Test. The Jonckheere-Terpstra test was used to compare non-normally distributed continuous variables among ordered categorical variable groups, followed by *post-hoc* multiple comparisons if differences were detected. Correlation analysis between ordered categorical variables or continuous variables and continuous variables was conducted using Spearman's correlation coefficient. Odds ratios (OR) were used to analyze the relationship between two categorical variables. The MacNemar test was employed to compare differences in the frequency of positive microbes between patients before and after recurrence. In the analysis of factors influencing recurrence, missing data for comorbidity and gallstone size information were imputed using the random forest model-based imputation method implemented in R's missForest package (Stekhoven and Bühlmann, 2012), with out-of-bag error rates from random forest used to report imputation results for complications and maximum gallstone diameter information. Logistic regression analysis was performed to evaluate factors influencing recurrence. Multivariate binary logistic regression analysis was conducted for variables that showed statistical significance in univariate analyses. All statistical tests were two-sided, and a *P*-value below 0.05 was considered statistically significant. Statistical analyses were performed using SPSS^®^ Statistics version 22 (IBM, Armonk, NY, USA) and R version 4.3.2.

## 3 Results

### 3.1 Baseline characteristics

Baseline characteristics of various clinical conditions are summarized in Table 1. Among 9,939 patients, females (5,299, 53.30%) were significantly more prevalent than males (4,640, 46.70%) (*P* < 0.001). The age distribution showed the highest proportion in the 60–74 age group (3,420, 34.41%), while the < 45 age group had the lowest proportion (1,488, 14.97%). Gallstone types were predominantly cholecystolithiasis (52.21%), followed by only EBD stones (40.17%). Patients without complications (6,731, 67.72%) was higher than those with complications (3,208, 32.28%). Maximum gallstone diameter, representing stone size, was available for 1,881 individuals, ranging from 2 to 60 mm, with 1,737 (92.34%) having sizes ≤ 20 mm. The most frequent sizes were 6 mm (247, 13.13%) and 10 mm (247, 13.13%) (Supplementary Figure S1A). Complications data were available for 2,181 patients, all of whom had at least one complication, with the highest proportion having one complication (878, 40.26%). As the number of complications increased, the number of patients decreased (Supplementary Figure S1B). Analysis of combinations of all patients' complication types revealed 317 unique combinations (Supplementary Table S3), with the top 10 combinations based on patient count, where only respiratory system diseases were most common (203, 9.31%), followed by hypertension alone (175, 8.02%) (Supplementary Figure S1C). Regarding recurrence, only 228 patients (2.30%) returned for diagnosis of recurrent gallstones, though this does not represent the recurrence rate as some recurrent patients may have sought treatment elsewhere without follow-up, categorized as “Unknown”. Subsequently, matching by age and gender was performed from the “Unknown” group to classify patients who did not recur as “No recurrent”. Among recurrent patients, recurrence occurred between 181 and 2,287 days, with a median of 616.00 days.

**Table 1:** Demographic and clinical characteristics.

**Characteristic**	**Designation**	**Data**	**Min-max**
Gender (*n* = 9,939)	Male	4,640 (46.70)	/
	Female	5,299 (53.30)	/
Age (*n* = 9,939)	/	64 (52, 75)	4-104
Age groups (*n* = 9,939)	< 45	1,488 (14.97)	/
	45-59	2,511 (25.26)	/
	60-74	3,420 (34.41)	/
	≥75	2,520 (25.36)	/
Types of gallstones (*n* = 9,939)	Cholecystolithiasis (C)	5,189 (52.21)	/
	Extrahepatic bile duct stones (EBD)	3,992 (40.17)	/
	Hepatolithiasis bile duct stones (HBD)	504 (5.07)	/
	C+EBD	183 (1.84)	/
	EBD+HBD	62 (0.62)	/
	C+HBD	4 (0.04)	/
	C+EBD+HBD	5 (0.05)	
Stone size (*n* = 1,181)	Maximum stone diameter (mm)	10 (6, 15)	2–60
Complications (*n* = 9,939)	No complications	6,731 (67.72)	/
	With complications	3,208 (32.28)	/
Comorbidities (*n* = 2,181)	Hypertension	918 (42.09)	/
	Respiratory diseases	874 (40.07)	/
	Coronary heart disease	474 (21.73)	/
	Diabetes	436 (19.99)	/
	Renal cyst	312 (14.31)	/
	Cerebral infarction	302 (13.85)	/
	Fatty liver	270 (12.38)	/
	Venous thrombosis	266 (12.20)	/
	Gallbladder adenomyomatosis	251 (11.51)	/
	Gallbladder polyp	211 (9.67)	/
	Malignant tumor	153 (7.02)	/
	Cirrhosis of the liver	90 (4.13)	/
Recurrence status	Recurrence	228 (2.30)	/
	No recurrence (Matched recurrence)	228 (2.30)	/
	Unkown	9,483 (95.4)	/
Days of recurrence	Days	616.00 (333.25, 1,000.25)	181–2,287

Categorical variables were presented as n (%). Normally distributed continuous variables were depicted by Mean ± SD, and nonnormally distributed continuous variables were represented as Q2 (Q1, Q3). Q1: First quartile; Q2: Second quartile; Q3: Third quartile.

### 3.2 Bile microbial characteristics in gallstone patients

**Figure 2:**
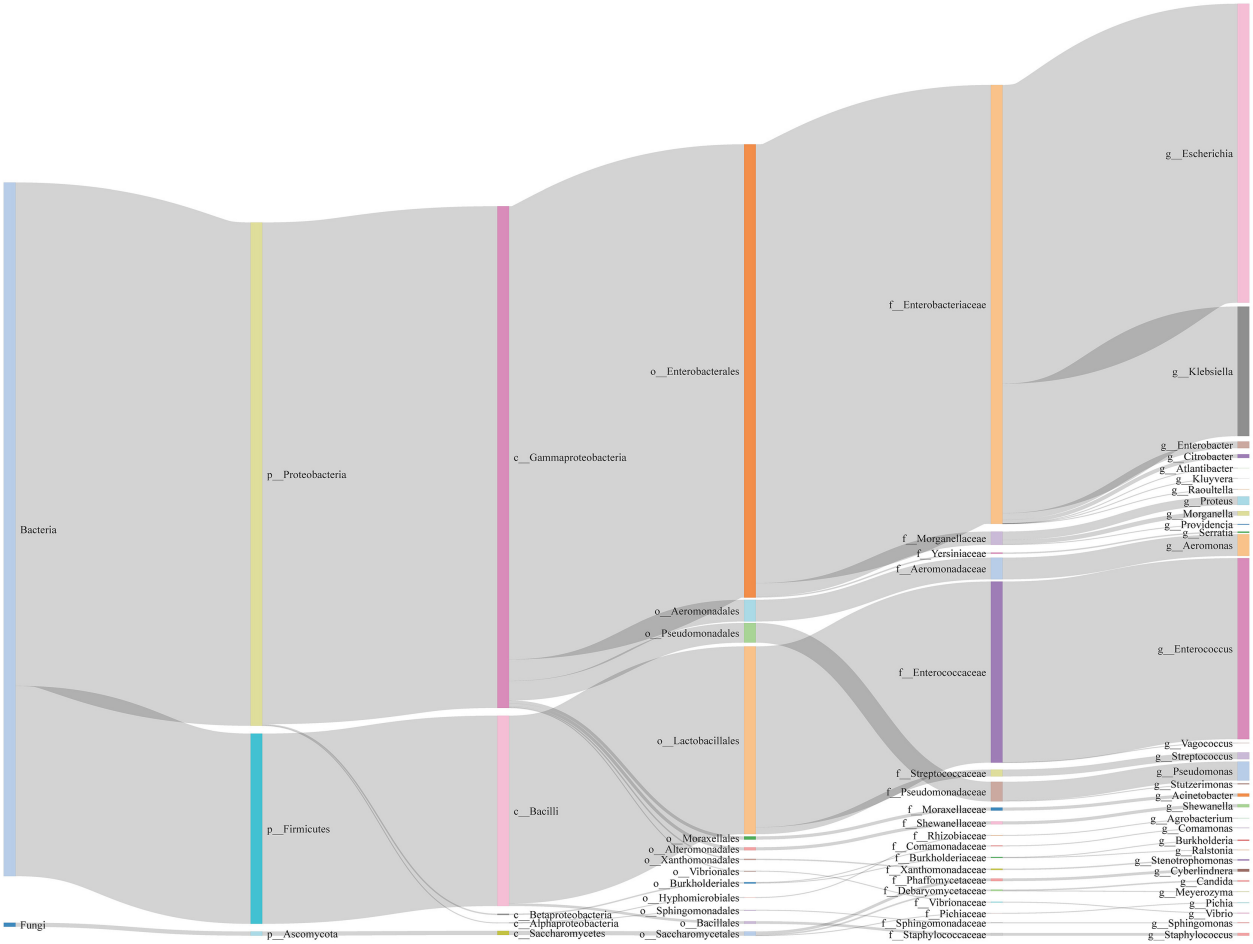
Sankey diagram of microbes in bile of all samples. We show the taxonomic ranks kingdom, phylum, class, order, family, genus.

In this study, 76 types of microbes were cultured from 5,153 (51.85%) patients, while no microbes were cultured from 4,786 (48.15%) patients (Supplementary Table S1). These 76 microbes included five fungi (*Candida parapsilosis, Candida tropicalis, Cyberlindnera jadinii, Meyerozyma guilliermondii, Pichia kudriavzevii*) and 71 bacteria, spanning three phyla (p__Ascomycota, p__Firmicutes, p__Proteobacteria), five classes (c__Alphaproteobacteria, c__Bacilli, c__Betaproteobacteria, c__Gammaproteobacteria, c__Saccharomycetes), 13 orders, 19 families, and 31 genera (Figure 2).

**Figure 3:**
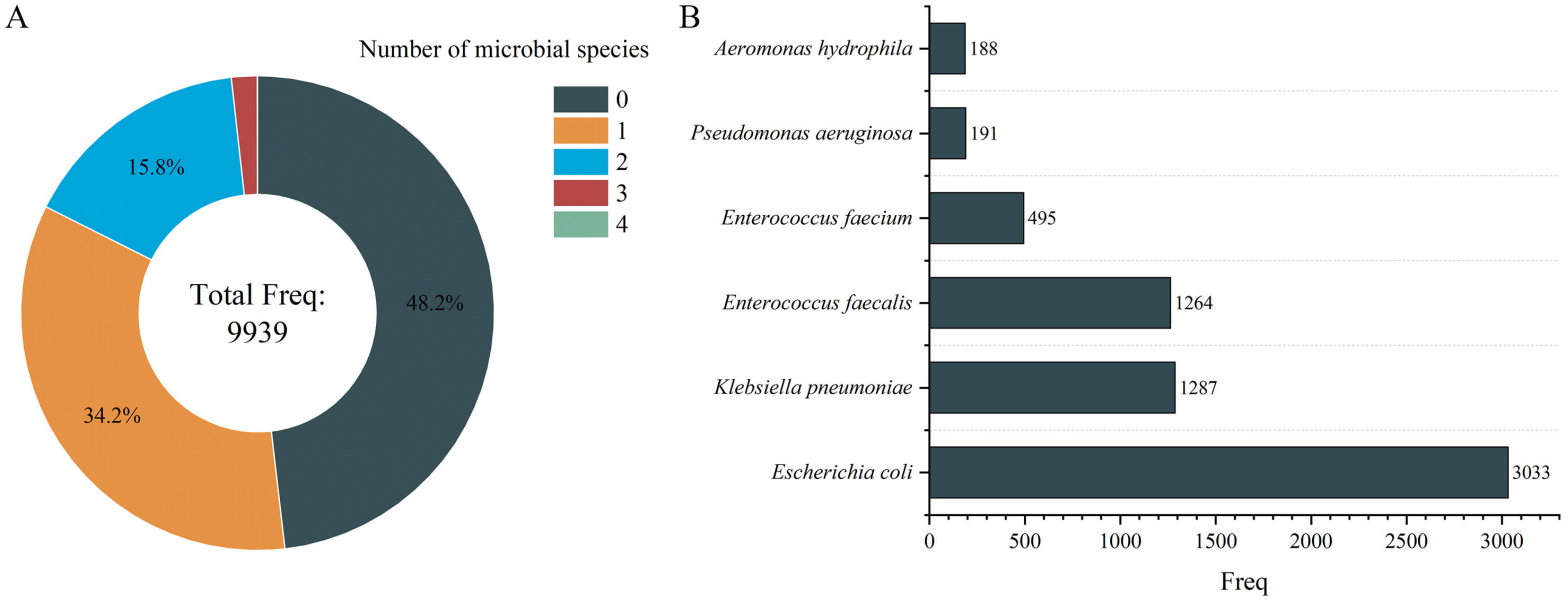
Number of microbial species distribution in gallstone individuals **(A)** and microbial distribution of dominant species **(B)**. Only the microbes with a frequency >100 individuals are displayed in **(B)**.

Regarding sample distribution, the number of people containing 1, 2, 3, and 4 species of microbes is 3,403, 1,573, 175, and 2, respectively, which shows that the higher the number of species, the lower the number of people located (Figure 3A). To identify dominant species, we analyzed the number of positive samples for each species, finding that *E. coli, Klebsiella pneumoniae*, and *Enterococcus faecalis* had the highest positive detection frequencies, with 3,033, 1,287, and 1,264 occurrences, respectively (Figure 3B).

### 3.3 Differences in clinical characteristics between microbial positive and negative groups

**Figure 4:**
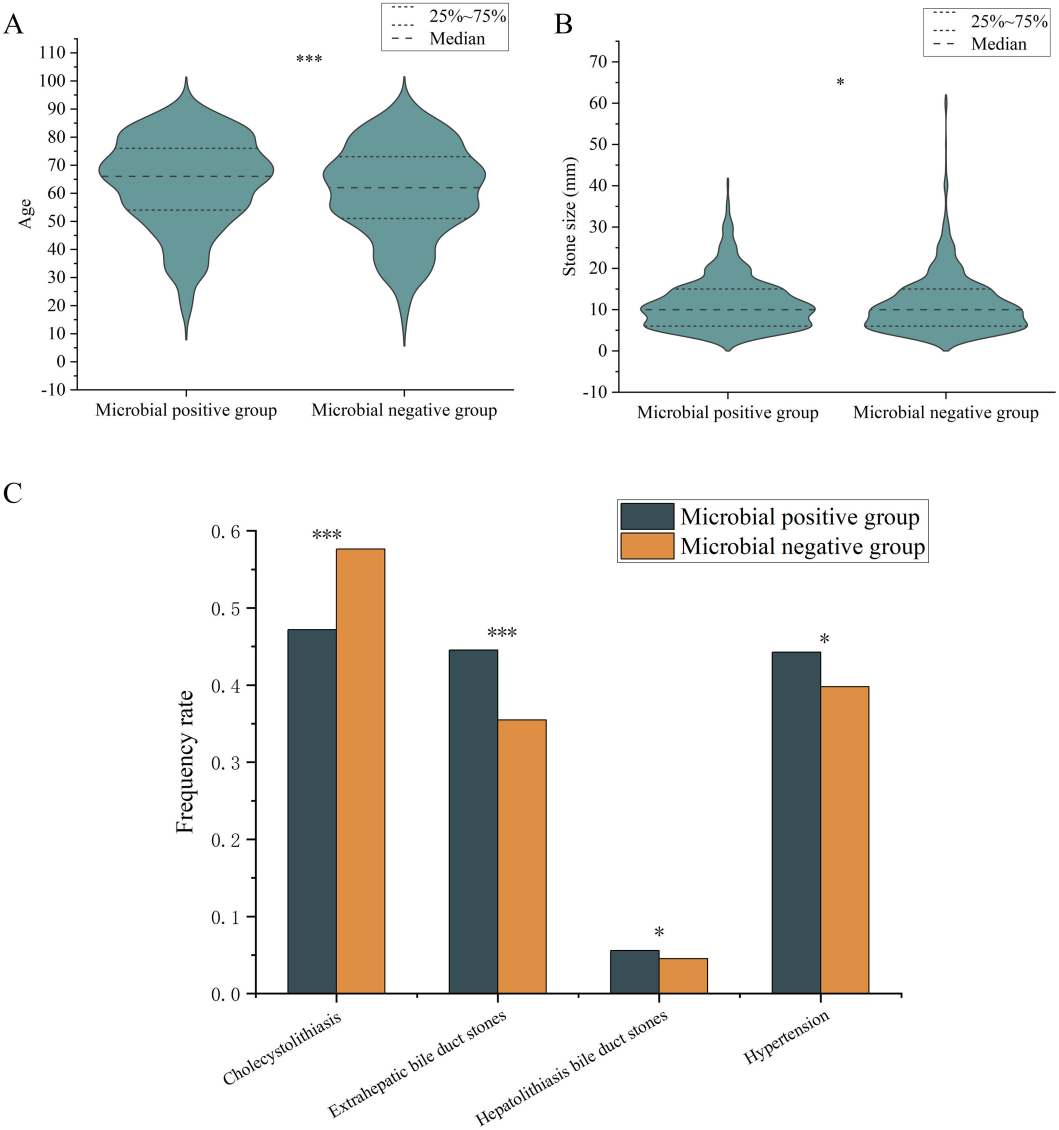
Differences in frequency rate of various variables between microbial positive and negative groups. **(A)** age; **(B)** stone sizes; **(C)** types of gallstones and comorbidities. “*” indicates *P* < 0.05; “***” indicates *P* < 0.001. Only comparisons with significant statistical difference (*P* < 0.05) are shown.

We explored whether there were significant differences in clinical characteristics between the Microbial Positive Group (MP group, *n* = 4,786) and the Microbial Negative Group (MN group, *n* = 5,153). The results showed that the MP group had significantly higher age (*P* < 0.001) and stone size (*P* = 0.043) compared to the MN group (Figures 4A, B). However, the effect size for stone size was small (r = 0.047), indicating that although the difference is statistically significant, the actual clinical impact of this difference is minimal. The occurrence frequencies of EBD stones (*P* < 0.001) and HBD stones (*P* = 0.015) were significantly higher in the MP group than in the MN group, while cholecystolithiasis were more frequent in the MN group (*P* < 0.001) (Figure 4C). Additionally, the incidence of hypertension was significantly higher in the MP group compared to the MN group (*P* = 0.034) (Figure 4C).

### 3.4 Differences in microbial diversity and positive detection rates across clinical characteristics

**Figure 5:**
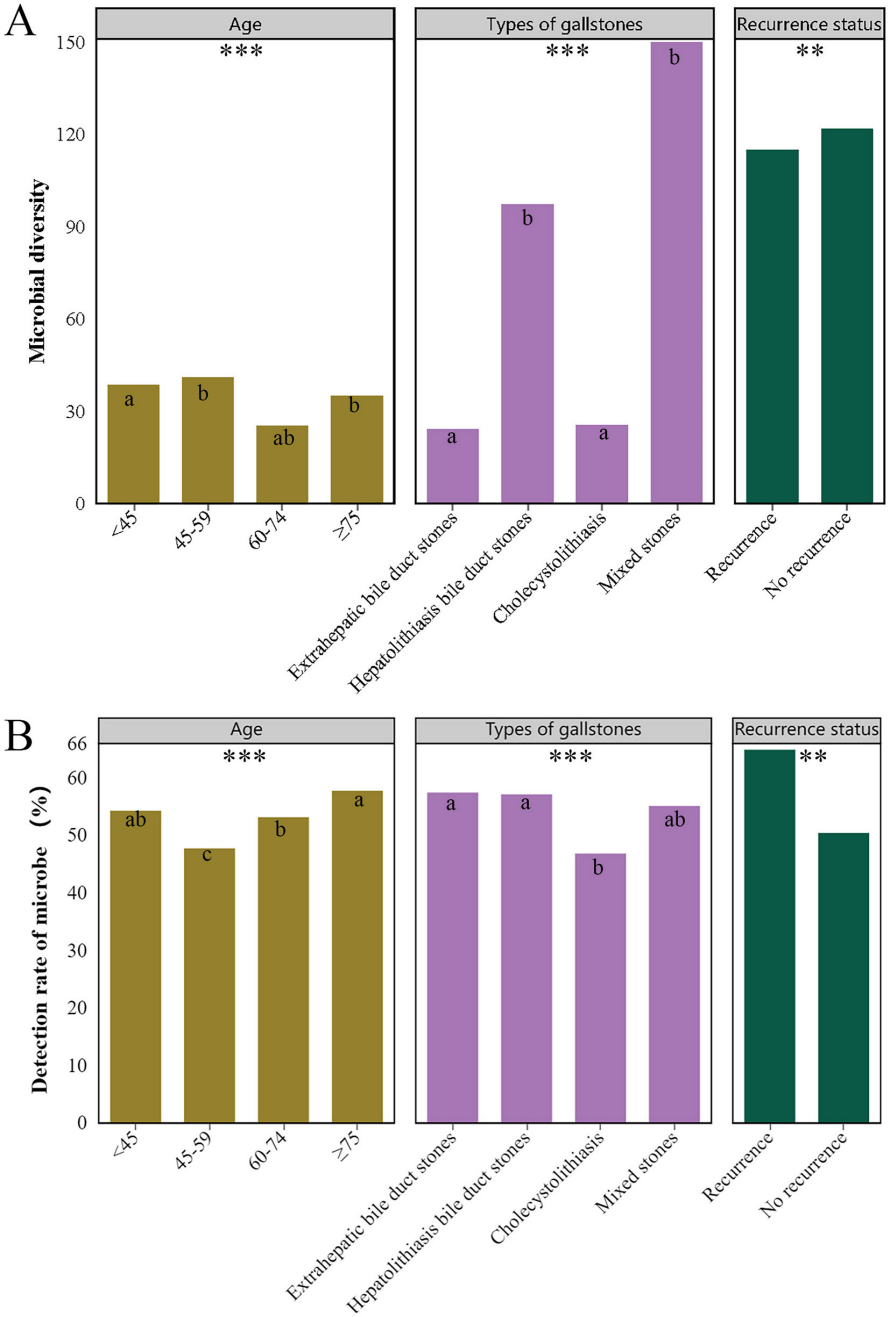
Differences in microbial diversity **(A)** and detection **(B)** rates across various clinical characteristics. Only clinical characteristics with significant differences are shown in the figure, namely different age groups, types of gallstones, and recurrence status. Different superscript letters (a, b and c) indicate significant differences. *** indicates *P* < 0.001, ** indicates *P* < 0.01.

We further compared microbial diversity and positive microbial detection rates across different clinical characteristics (gender, age, gallstone types, presence of complications, recurrence status). The results indicated significant differences in microbial diversity and positive detection rates among different age groups (*P* < 0.001), gallstone types (*P* < 0.001), and recurrence status (*P* = 0.012; *P* = 0.002) (Figure 5). Specifically, microbial diversity was significantly higher in the 45–59 age group, patients with HBD stones and mixed stones, and no recurrent group. The positive detection rate was significantly higher in patients aged ≥75, those with EBD stones and HBD stones, and in the recurrent group.

### 3.5 Differences in microbial diversity and positive detection rates across clinical characteristics

Based on clinical variables, we categorized the patients into male and female groups, four different age groups, groups with four different gallstone types, groups with or without complications, and recurrence vs. non-recurrence groups, to explore the differences in specific microbes across these classifications. The results showed that Streptococcus sanguinis exhibited a significant difference between males and females (*P* = 0.036), but its frequency was very low, with only five detections among 5,299 females and none among 4,640 males.

**Figure 6:**
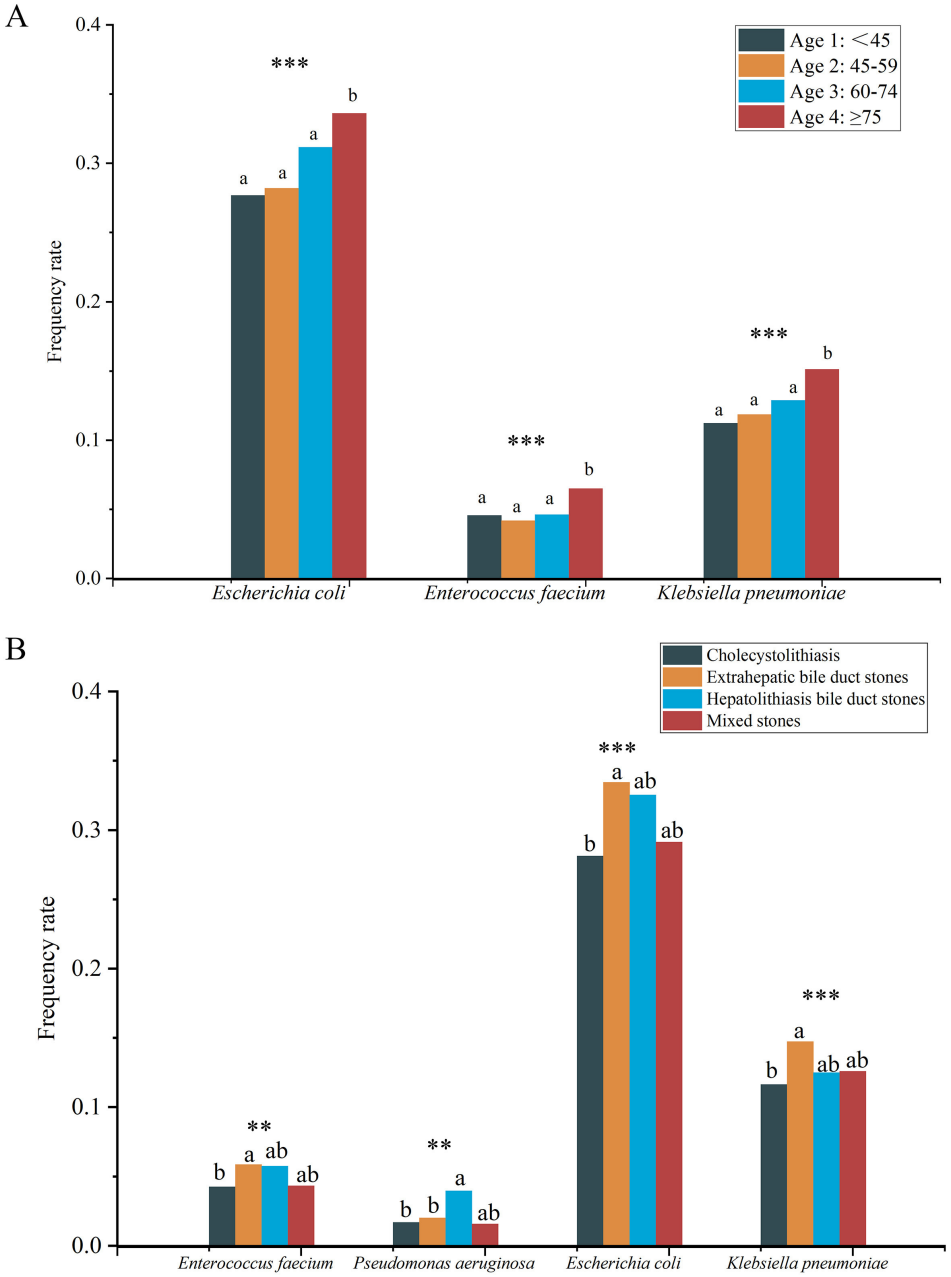
Differences in frequency rate of specific microbes among four different age groups **(A)** and different types of gallstones **(B)**. Different lowercase superscript letters indicate significant differences among different groups (*P* < 0.05). *** indicates *P* < 0.001, ** indicates *P* < 0.01.

The positive frequencies of *E. coli* (*P* < 0.001), *Enterococcus faecium* (*P* < 0.001), and *K. pneumoniae* (*P* < 0.001) differed significantly among the four groups, and all of them were significantly higher in the ≥75 years age group than in the other three groups (Figure 6A).

Significant differences were also found in the positive frequencies of *E. coli* (*P* < 0.001), *K. pneumoniae* (*P* < 0.001), *E. faecium* (*P* = 0.004), and *Pseudomonas aeruginosa* (*P* = 0.004) among different gallstone types (Figure 6B). Specifically, the positive frequencies of *E. faecium, E. coli*, and *K. pneumoniae* were significantly higher in the EBD group compared to the cholecystolithiasis group, while P. aeruginosa had a significantly higher positive frequency in the HBD group compared to the EBD and cholecystolithiasis groups. It is noteworthy that no significant differences were found in the frequencies of any microbes between the groups with or without complications, and between the recurrence and no recurrence groups.

To further explore the association between microbes and various clinical variables, we performed odds ratio (OR) analysis. The results indicated that 15 microbes were significantly associated with clinical variables (Supplementary Figure S2). While the data analysis revealed meaningful correlations, it is important to note that the frequencies of these microbes were relatively low in the total sample (2–26 occurrences), which might introduce random effects and potentially affect the robustness and generalizability of our findings.

### 3.6 Microbes and recurrence

The aforementioned analysis found that microbial diversity was significantly reduced in the recurrence group compared to the no recurrence group. However, no significant differences in the frequencies of specific microbes were observed between these two groups, and there was no significant difference in recurrence rates between the MN and MP groups.

**Figure 7:**
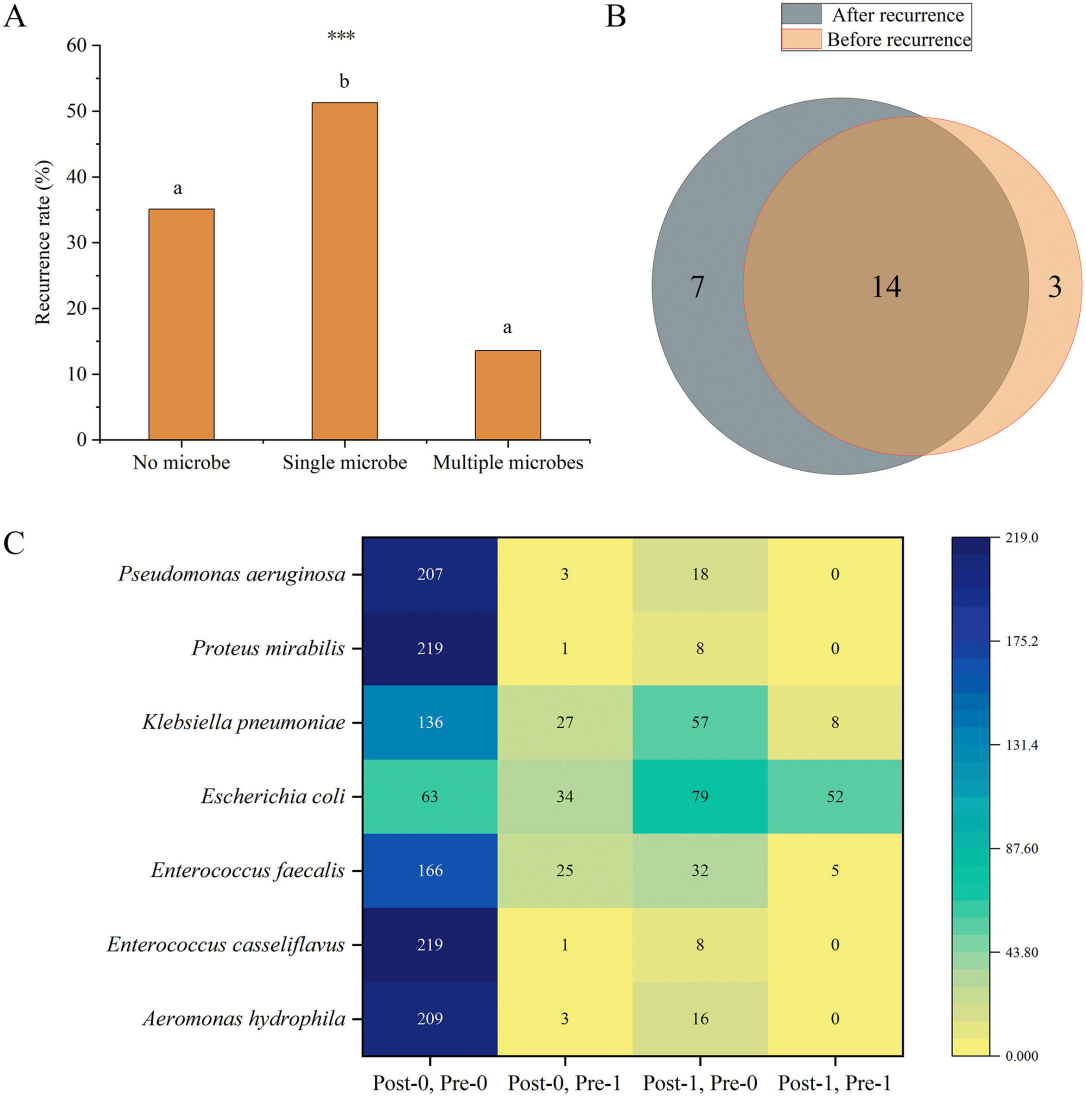
**(A)** Differences in recurrence frequencies among the groups with no, single, and multiple microbes' groups. Different lowercase superscript letters indicate significant differences among different groups (*P* < 0.05). *** indicates *P* < 0.001. **(B)** Venn diagram of the number of microbial species present before and after recurrence. **(C)** Distribution of the frequencies of microbes with significant differences before and after recurrence. The heatmap shows the distribution of frequencies for seven microbes under different conditions before (Pre) and after (Post) recurrence. The vertical axis lists the microbial species, while the horizontal axis represents different condition combinations. Post-0, Pre-0 indicates the number of patients who were negative for the microorganism both before and after recurrence; Post-0, Pre-1 indicates the number of patients who were positive before recurrence and negative after recurrence; Post-1, Pre-0 indicates the number of patients who were negative before recurrence and positive after recurrence; Post-1, Pre-1 indicates the number of patients who were positive both before and after recurrence. Numerical labels indicate specific frequency counts, and the shading of the color represents the magnitude of the counts, with darker colors indicating higher frequencies.

To further investigate the relationship between the number of microbial species and recurrence, we divided the patients into no microbe group, single microbe group, and multiple microbe group (2 or 3 microbial species) and compared the differences in recurrence frequencies among these groups. The results showed that the recurrence rate in the single microbe group was significantly higher than in the other two groups (*P* < 0.001) (Figure 7A).

Moreover, we further analyzed the changes in microbes before and after recurrence in patients with recurrence. Among the 228 recurrence patients, 17 microbes were detected before recurrence, and 21 microbes were detected after recurrence. 14 microbes were common to both before and after recurrence (Figure 7B). We primarily focused on microbes whose frequencies showed significant changes before and after recurrence. The results revealed that the frequencies of seven microbes (*Aeromonas hydrophila, P* = 0.004; *E. casseliflavus, P* = 0.039; *E. faecium, P* = 0.013; *E. coli, P* < 0.001; *K. pneumoniae, P* = 0.002; *Proteus mirabilis, P* = 0.039; *P. aeruginosa, P* = 0.001) were significantly different before and after recurrence, and all of these microbes were detected in more patients after recurrence (Figure 7C).

**Figure 8:**
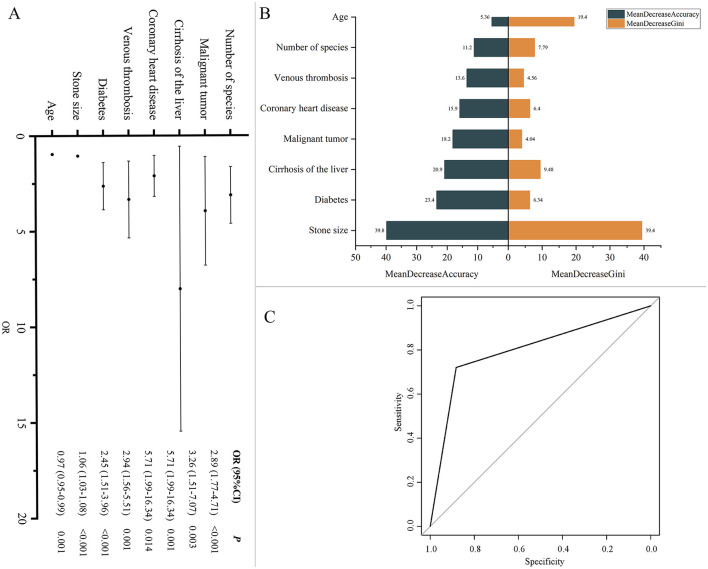
**(A)** Factors influencing recurrence. Multivariate binary logistic regression analysis was conducted to calculate odds ratios (ORs) and 95% confidence intervals (CI), assessing factors associated with recurrence. **(B)** Model variable importance based on MeanDecreaseGini and MeanDecreaseAccuracy. **(C)** ROC Curve for recurrence prediction model. ROC curve illustrating the performance of the model in terms of sensitivity and specificity.

To identify factors influencing recurrence, we conducted univariate and multivariate regression analyses. Univariate regression analysis (including all clinical variables and 76 microbes) initially screened the factors affecting recurrence (Supplementary Table S4). Using these variables, along with sex and age as basic variables, we further performed multivariate binary logistic regression analysis (Supplementary Table S5). The results indicated that age (OR, 0.97; 95% CI, 0.95–0.99, *P* = 0.001), stone size (OR, 1.06; 95% CI, 1.03–1.08, *P* < 0.001), diabetes (OR, 2.45; 95% CI, 1.51–3.96, *P* < 0.001), venous thrombosis (OR, 2.94; 95% CI, 1.56–5.51, *P* = 0.001), cirrhosis of the liver (OR, 5.71; 95% CI, 1.99–16.34, *P* = 0.001), malignant tumors (OR, 3.26; 95% CI, 1.51–7.07, *P* = 0.003), coronary heart disease (OR, 5.71; 95% CI, 1.99–16.34, *P* = 0.014), and the microbial species (OR, 2.89; 95% CI, 1.77–4.71, *P* < 0.001) were significant predictors of recurrence (Supplementary Table S5, Figure 8A). We further used these variables to construct a random forest model. The MeanDecreaseGini and MeanDecreaseAccuracy provided by the random forest model were used to indicate the importance of variables within the model (Figure 8B). The results showed that stone size contributed the most to model accuracy and purity. The model's accuracy, precision, recall, F1 score, and ROC-AUC values (Figure 8C) were 0.801, 0.721, 0.860, 0.784, and 0.862, respectively, indicating that the model is an excellent predictive tool with good performance and high predictive power in forecasting recurrence.

## 4 Discussion

This study analyzed the clinical characteristics and bile microbiology of 9,939 patients with gallstones. The results showed that there were significantly more females than males in the study population, and the age distribution was mainly between 60 and 74 years. Gallbladder stones were the most common type, accounting for over half of the cases. Microbial cultures were positive in 5,153 patients (51.85%), yielding 76 microbes (5 fungi and 71 bacteria). We further analyzed the data from various perspectives, including differences in clinical characteristics between MP and MN groups, variations in microbial diversity and positivity rates across different clinical characteristics, differences in specific microbes with varying clinical features and their correlation with clinical variables, and the relationship between microbes and recurrence. These analyses revealed several important clinical and microbial features, providing a foundation for further exploration of recurrence mechanisms and clinical management.

Among the 9,939 gallstone patients, females significantly outnumbered males, and the majority of patients were in the 60–74 age group. A recent nationwide study on the age, gender, geographic, and clinical differences in gallstone disease in China found that, regardless of anatomical location, the prevalence of gallstones significantly increases with age (*P* < 0.001), with the age-standardized prevalence higher in females than in males (5.41% vs. 4.85%, *P* < 0.001) (Song et al., 2022). Another study reported that the prevalence of gallstone disease increases with age, with male and female prevalence rates of 7.1% and 10.4%, respectively (Zhang et al., 2022). Our findings are consistent with previous studies, indicating that gallstones are more common in the elderly, with a higher prevalence in females. Regarding gallstone types, gallbladder stones were the most prevalent, reflecting their common occurrence in clinical practice.

Bile in healthy individuals is typically sterile; however, under certain conditions, such as bile duct obstruction, bacteria can proliferate in the stagnant bile. Earlier hypotheses suggested that different bacterial species might increase the formation of gallstones and biliary diseases through specific enzyme activities or biofilm production; several studies have proposed their association with bacterial infections (Kosowski et al., 1987; Lazcano-Ponce et al., 2001; Sattar et al., 2007). However, the exact mechanism of this phenomenon remains unclear (Hoogerwerf and Soloway, 1999; Vitetta et al., 2000). The role of bacteria in biliary disease formation is primarily considered in relation to cholelithiasis, which accounts for 90% of global cholelithiasis cases (Gomes et al., 2009). The involvement of bacteria in the formation of biliary stones is still debated. The formation of gallstones is attributed to bacteria originating from the intestine that ascend to the bile ducts due to various predisposing conditions (Englesbe and Dawes, 2005). *Escherichia coli* and other gut bacteria, such as *Enterococcus* spp., are the most common bacteria in these infections. These bacteria can adhere to and colonize the biliary epithelium using the same surface proteins known as adhesins (Ljungh and Wadström, 2002). Factors such as bacterial mucus, antibiotic resistance in bile, and biofilm formation by bacteria are considered crucial in gallstone formation. Long-term exposure to bile salts is known to induce biofilm formation in enteric pathogens from the Enterobacteriaceae family, such as *Salmonella* and *Shigella* spp., as well as emerging pathogens like *E. coli, K. pneumoniae, Enterococcus* spp., and *Clostridium* spp. (Tsuchiya et al., 2018). Furthermore, biofilm formation and anaerobic energy metabolism are considered potential microbial mechanisms involved in gallstone formation. The bacterial composition of the stones and identified enteric bacteria, such as *Enterobacter* spp., *Enterococcus* spp., *Escherichia* spp., *Klebsiella* spp., and *Salmonella* spp., are contributors to gallstone formation (Ballal et al., 2001; Sattar et al., 2007; Gomes et al., 2009; Joo and Otto, 2012). In our study, microbial cultures were positive in 5,153 patients (51.85%), with *E. coli, K. pneumoniae*, and *E. faecalis* being the most common microbes. This finding aligns with other studies identifying common pathogens associated with gallstone-related infections (Ballal et al., 2001; Sattar et al., 2007; Gomes et al., 2009; Lévay et al., 2013; Tajeddin et al., 2016; Pagani et al., 2023). *E. coli* is a common human pathogen, a Gram-negative, facultative anaerobic rod from the Enterobacteriaceae family, capable of causing various infections in different anatomical sites and invading the bile ducts during cholestasis, contributing to gallstone formation (Tajeddin et al., 2016; Blesl and Stadlbauer, 2021). *K. pneumoniae* was first reported in Taiwan in 1980 and gradually became the leading pathogen of pyogenic liver abscess (PLA) in Asia, commonly found in East Asian populations, often associated with diabetes and gallstones (Serraino et al., 2018; David et al., 2021). *E. faecalis* is a Gram-positive bacterium found in the gastrointestinal tracts of humans and animals, classified as a facultative anaerobe, with dual metabolic lifespans that enhance its robustness and stress resistance, and is associated with human infections, particularly in immunocompromised individuals, causing diseases such as endocarditis, meningitis, pneumonia, peritonitis, visceral abscesses, urinary tract infections, and sepsis (Boeder et al., 2024). Studies have shown that *E. faecalis* is abundant in patients with chronic hepatobiliary diseases and may induce hepatic tumorigenesis (Xie et al., 2016; American Association for Cancer Research, 2021; Große et al., 2021; Iida et al., 2021; Awoniyi et al., 2023).

This study is the first to compare the clinical characteristics of bile MP and MN groups in patients with gallstones. It was found that patients in the MP group had significantly higher age, and incidence of hypertension compared to the MN group, indicating that the presence of microbes may be associated with more severe conditions. In addition, the frequency of bile duct stones was significantly higher in the MP group than in the MN group whereas the opposite was true for cholecystolithiasis (*P* < 0.001), suggesting that microbes play different roles in the formation and development of stones at different sites (Lee et al., 2023), and that the high frequency of bile duct stones may be related to the complex anatomy of the biliary system and its greater susceptibility to microbial infection. In contrast, the formation of cholecystolithiasis may be more influenced by the environment of the gallbladder itself.

Microbial diversity was particularly enriched in the 45–59 age group, as well as among patients with HBD and mixed stones. The relatively stable immune system activity and biliary microenvironment in patients aged 45–59 may support a diverse microbial community, potentially reducing pathogen colonization and infection. The complex structure of intrahepatic bile ducts provides various ecological niches for the coexistence of multiple microbes, while mixed stones may reflect the interaction between the gallbladder and bile ducts, promoting microbial diversity (Feng et al., 2022). The positive detection rate of microbes was significantly higher in patients aged ≥75, and those with EBD and HBD stones. The increased positive detection rate in elderly patients (≥75 years) may be due to immune system decline and a higher likelihood of chronic diseases, which heighten infection risk (Lee et al., 2023). The high microbial positivity rate in bile duct stones might be linked to the complex microenvironment within the bile ducts and their susceptibility to infections, making them a favorable habitat for microbes.

We further analyzed the differences in specific microbes across various clinical characteristics and their correlations with clinical variables. The results showed that the positive frequencies of *E. coli, E. faecium*, and *K. pneumoniae* were significantly higher in the ≥75 age group compared to other age groups, which may be related to the declining immune function in elderly patients. The adaptability and antibiotic resistance of *E. coli* within the biliary tract make it more likely to proliferate in older patients; meanwhile, *E. faecium* is known for its antibiotic resistance and is commonly associated with nosocomial infections (Boeder et al., 2024). The increased risk of infection in elderly patients may be further exacerbated by frequent hospitalizations and the use of broad-spectrum antibiotics. *K. pneumoniae* is associated with a variety of infections, including pneumonia, urinary tract infections, and biliary infections (Xie et al., 2016; American Association for Cancer Research, 2021; Große et al., 2021; Awoniyi et al., 2023; Boeder et al., 2024), and its high positive frequency in elderly patients is particularly notable, possibly due to the higher rates of hospitalization and invasive procedures (e.g., catheterization and biliary stenting) in this population, which increase the risk of infection. These findings highlight the importance of heightened vigilance in the prevention and management of biliary infections in elderly patients. In different types of gallstones, the positive frequencies of *E. faecium, E. coli*, and *K. pneumoniae* were significantly higher in the EBD group compared to the cholecystolithiasis group, which may be attributed to the open structure of the extrahepatic bile ducts, increasing the risk of infection. In the HBD group, the positive frequency of *P. aeruginosa* was significantly higher than in the EBD and cholecystolithiasis groups, potentially due to its adaptability to the complex bile duct structure and liver environment, as well as its high antibiotic resistance. Studies have shown that 30% of cholesterol gallstones can be cultured for strains that secrete β-glucuronidase and phospholipase A2, with *P. aeruginosa* exhibiting the highest β-glucuronidase activity, which may be a key factor in cholesterol gallstone formation (Peng et al., 2015). Additionally, the results of the culture method revealed that biofilm-forming bacteria (*P. aeruginosa, E. coli, K. pneumoniae, Enterococcus spp*., and *Acinetobacter* spp.) coexist in various combinations and are the main bacteria associated with cholelithiasis (Tajeddin et al., 2016; Pagani et al., 2023). These organisms can form robust biofilms, enhancing colonization and antibiotic resistance, which presents challenges in treatment. This suggests the need for increased monitoring and targeted therapy against these species, particularly in elderly patients with compromised immune function and those with bile duct stones. Furthermore, our study explored the associations between microbes and various clinical variables using OR analysis. Although we identified significant associations between 15 microbes and specific clinical variables in a sample of 9,939 cases, it is important to note that the detection frequency of each microbe was relatively low. We believe that the current findings may be influenced by chance and bias, limiting the value of further analysis and discussion. Therefore, we do not recommend an in-depth exploration of these associations based on the existing data. Instead, future research should focus on microbes with greater clinical significance and higher detection frequencies.

Gallstone disease recurrence is a common and serious issue. Existing studies indicate that patients with recurrent gallstones often have poorer clinical outcomes, which is often accompanied by a higher complication rate and a more frequent need for surgical intervention, especially an increase in the incidence of gangrenous cholecystitis during the COVID-19 pandemic (De Simone et al., 2022). In this study, microbial diversity was found to be lower in the recurrence group compared to the no recurrence group. The rich microbial community in the non-recurrent group may fight against pathogenic bacteria through mechanisms such as competitive inhibition and production of antimicrobial substances, preventing the overgrowth of pathogenic bacteria and the re-formation of stones and reducing the risk of recurrence (Lee et al., 2023). Our findings are consistent with previous reports. In the study by Choe et al., which examined the microbial characteristics and bile composition of patients with recurrent common bile duct (CBD) stones after endoscopic treatment using 16S rRNA sequencing, the microbial richness was significantly lower in the recurrence group, and microbial evenness was also reduced. Differences in the overall microbial community were observed between the recurrence and non-recurrence groups (Choe et al., 2021). Additionally, another study based on 16S rDNA gene sequencing also demonstrated a significant reduction in biliary microbial diversity in patients with recurrent CBD stones (Tan et al., 2022). These findings further support the critical role of microbial diversity in the recurrence of gallstone disease. In our study, the number of patients with positive microbial cultures was significantly higher in the recurrence group, which may be related to persistent infections and residual microbes. Patients with recurrent gallstones may experience ongoing biliary infections, or residual microbes from an initial infection that was not fully eradicated, thereby increasing the risk of stone recurrence. Further analysis revealed that the recurrence rate in singe microbe group was significantly higher than in groups of no microbe and multiple microbes. This suggests that the presence of a single pathogen may increase the risk of gallstone recurrence by influencing biofilm formation and inducing persistent inflammation. In contrast, the coexistence of multiple microbes may reduce the risk of recurrence by promoting microbial competition and ecological balance, which can inhibit the overgrowth of certain pathogenic bacteria. The lower recurrence rate in the no microbe group may be due to a reduced inflammatory response and biofilm formation, processes that are closely associated with stone formation and recurrence. In summary, these results indicate that microbial diversity plays a key role in the recurrence of gallstone disease, offering new insights into the prevention and treatment of gallstone recurrence.

Seven microbes (*A. hydrophila, E. casseliflavus, E. faecium, E. coli, K. pneumoniae, P. mirabilis, P. aeruginosa*) showed significant frequency differences before and after recurrence, and all seven were present in more patients after recurrence, suggesting they may play a crucial role in gallstone recurrence. Changes in microbial frequency before and after recurrence may reflect dysbiosis or suboptimal antimicrobial treatment during the patient's course of therapy. As previously mentioned, *Enterococcus faecium, E. coli*, and *K. pneumoniae* are dominant microbial species in the bile of gallstone patients, and their frequencies differed significantly before and after recurrence. The high adaptability and persistence of *E. coli* and *K. pneumoniae* in the biliary tract may promote cholesterol crystal formation, exacerbating stone recurrence. Additionally, their ability to form biofilms increases the difficulty of clearing infections. *E. casseliflavus* and *E. faecium* are common enterococci with pathogenicity and antibiotic resistance (Narciso-Schiavon et al., 2015; Britt and Potter, 2016). *E. casseliflavus* has a high affinity for the biliary system and is prone to causing biliary and liver infections (Yoshino, 2023). *E. faecium* exhibits resistance to multiple antibiotics, including vancomycin and oxazolidinone (Boeder et al., 2024), which may increase the difficulty of infection control in the recurrence group. In 2021, an *E. faecium* strain carrying an oxazolidinone resistance gene was isolated from the bile of a common bile duct stone patient in Shenzhen, China, highlighting the complexity and resistance of this pathogen within the biliary system (Deng et al., 2023). The high pathogenicity and antibiotic resistance of these bacteria may exacerbate biliary inflammation, promoting the formation and recurrence of gallstones, particularly as *E. faecium*'s resistance complicates the eradication of infections in recurrent patients. *A. hydrophila* is an emerging pathogen found widely in aquatic environments and has been reported to cause various severe infections, such as gastroenteritis, skin infections, peritonitis, bacteremia, meningitis, and necrotizing fasciitis (Citterio and Francesca, 2015). In one case report, a 72-year-old female patient with gallstones and hypertension died from necrotizing fasciitis caused by *A. hydrophila* following laparoscopic cholecystectomy (Janjua et al., 2024). Another case involved a 72-year-old elderly patient with gallstones and rheumatoid arthritis on immunosuppressive therapy (tocilizumab) who developed septicemia and acute suppurative cholangitis due to *A. hydrophila* (Okumura et al., 2011). These cases emphasize the high pathogenicity of *A. hydrophila* in postoperative infections and its severe impact on prognosis. Its broad virulence factors may trigger or exacerbate biliary inflammation in gallstone patients, thereby promoting stone recurrence. *P. mirabilis* is known for its ability to produce urease, which breaks down urea to produce ammonia, alkalinizing the local environment and facilitating stone formation (Armbruster and Mobley, 2012; Norsworthy and Pearson, 2017). Its role in gallstone recurrence may be similar, promoting recurrence by altering the local environment. *Pseudomonas aeruginosa*, as a highly resistant pathogen capable of forming biofilms, has been widely recognized for its role in biliary infections and gallstone formation (Peng et al., 2015; Tajeddin et al., 2016; Pagani et al., 2023). The increase in this bacterium after gallstone recurrence may be related to changes in the biliary environment and immune status, and its resistance adds to the challenge of controlling recurrence. In this study, we have compiled a table (Supplementary Table S6) comparing the microorganisms identified in our research with those associated with cholelithiasis as reported in the published literature. This comparison aims to provide a clearer illustration of the relationship between our findings and the existing body of scientific knowledge.

We conducted regression analysis to identify factors influencing gallstone recurrence, including the number of microbial species, age, stone size, diabetes, venous thrombosis, cirrhosis of the liver, malignant tumors, and coronary heart disease. Studies have shown that older age, larger stone size, the presence of diabetes and other factors are associated with an increased risk of gallstone formation and recurrence (Konstantakis et al., 2017; Muratori et al., 2017; Song et al., 2020; Binh et al., 2022; Sun et al., 2022). By integrating microbial data with clinical variables, we developed a machine learning model that performed well in predicting recurrence (ROC-AUC = 0.862). This multifactorial model offers a more comprehensive approach compared to traditional single-factor analyses, enhancing the accuracy and reliability of recurrence predictions. Future research could further optimize this model by incorporating additional potential predictive variables and validating its applicability and stability across different populations. The results of this study underscore the importance of early identification and management of high-risk patients, particularly those with a high risk of recurrence. We recommend developing differentiated treatment strategies for various types of gallstones, enhancing preoperative assessment, optimizing antimicrobial therapy, and improving postoperative monitoring and early intervention, especially in elderly and immunosuppressed patients.

Due to clinical diagnostic needs, our institution currently employs culture-based methods for bile microbial identification. Although the number of cultivable microbial species is limited, culture methods possess several irreplaceable advantages: (1) Culture methods can directly isolate pathogenic microorganisms from clinical samples for subsequent identification and antimicrobial susceptibility testing. This remains the only approach that enables the isolation of live pathogens for further susceptibility testing, which is crucial for guiding appropriate antimicrobial therapy. (2) Culture methods allow for the differentiation between live and dead bacteria, which is critical for assessing microbial activity and physiological state. Although sequencing techniques offer higher sensitivity, they cannot effectively distinguish between viable cells and free DNA. (3) Culturing enables preliminary microbial identification through visual observation of colony morphology, which is essential for rapid diagnosis and timely treatment, whereas sequencing typically requires longer analysis times and is not suited for urgent clinical decision-making. (4) The high sensitivity of sequencing can sometimes result in false positives due to contaminants. In contrast, the unique advantage of culture methods is their focus on viable microorganisms, reducing the likelihood of false positives. Moreover, given the large sample size in our study, the microbial identification results obtained through culturing are highly reliable and reproducible. Numerous high-quality studies have also successfully utilized culture-based methods to explore the relationship between microorganisms and clinical characteristics (Hooton et al., 2013; Cobo et al., 2017; Kwong et al., 2018). Therefore, despite the limitations of culture-based methods in capturing the full spectrum of microbial diversity, they remain an invaluable tool in clinical microbiology for providing actionable information on the bile microbiota in gallstone patients. While we acknowledge that more advanced microbiological techniques, including anaerobic culture methods, could provide a more comprehensive analysis of microbial composition, the objective of this study was to perform an initial exploration of microbial characteristics in bile from patients with gallstone disease. Therefore, we adopted standard microbial culture methods, which allow for rapid and reliable identification of microorganisms in bile. In future studies, we plan to incorporate more advanced techniques to achieve a deeper understanding of the microbial communities.

This study has several limitations: (1) Data were missing, with a high rate of missing information for gallstone size and comorbidity samples. (2) Recurrence rates could not be accurately calculated. Not all persons were followed up due to the difficulty of follow-up and the large sample size. (3) Recurrence time was influenced by patient subjective factors. The number of days to recurrence in this study is the number of cases of recurrence found in the second visit of the patients on their own initiative, and we could not accurately obtain the number of days to recurrence of the patients unless they were frequently monitored for review. (4) There may be errors in non-recurrent patients. Non-recurrent patients were followed up only by telephone and there may have been asymptomatic recurrences that went undetected. Future research should further explore the complex pathophysiological processes and multifactorial interactions between gallstones and bile microbes. By integrating culture and sequencing data, a more accurate microbial ecological model could be constructed, providing more comprehensive and in-depth etiological evidence. Additionally, the development of personalized medicine should be emphasized, incorporating clinical data, microbiological information, and genomic data to design targeted antimicrobial therapies and individualized treatment plans, as well as recurrence risk prediction models based on multivariable data.

## 5 Conclusion

This study systematically analyzed the baseline characteristics, microbial profiles, and the relationship between microbiota and clinical features, particularly with recurrence, in gallstone patients. Among the 9,939 patients, 5,153 were able to culture 76 microbial species. *E. coli, K. pneumoniae*, and *E. faecalis* were the most common microbes. Certain specific microbes were significantly associated with clinical characteristics such as age and gallstone type. Compared to the no recurrence group, the recurrence group exhibited significantly reduced microbial diversity. In recurrent patients, the frequency of seven specific microbes showed significant differences before and after recurrence, with all appearing more frequently after recurrence. Factors such as the number of microbial species, age, stone size, and diabetes were important predictors of gallstone recurrence. These findings provide valuable insights for the prevention, diagnosis, and treatment of gallstones.

## Data Availability

The original contributions presented in the study are included in the article/Supplementary material, further inquiries can be directed to the corresponding author.

## Supplementary material

Supplementary Figure S1 Stone size and comorbidity information. **(A)** Distribution of stone sizes. This figure clearly shows the range and concentration trend of stone sizes. The horizontal axis represents patient density, and the vertical axis represents stone size. The wider the section, the more patients fall within that stone size range. **(B)** Number and proportion of comorbidities. Each patient belongs to only one category. For example, the red section indicates that 40.3% of patients have only one comorbidity, while the blue section shows that 29.1% of patients have two comorbidities. Only a very small portion of patients have six or seven comorbidities simultaneously. **(C)** Categories and proportions of comorbidity combinations. Each patient belongs to only one category. Each patient belongs to only one category, meaning each patient is counted under one comorbidity combination. This classification helps identify the most common comorbidity combinations and their distribution. For example, gallstone patients with only respiratory system diseases represent the largest group (9.31%), followed by those with only hypertension (8.02%).

Supplementary Figure S2 Correlation heatmap of categorical variables. The figure displays only significant correlations (*P* < 0.05), with white areas indicating no significant difference. The reference category is the absence of the event. For instance, the reference category for recurrence is no recurrence.

Supplementary Table S1 Clinical data.

Supplementary Table S2 Bile microbes of patients after recurrence.

Supplementary Table S3 Types and numbers of comorbidity combinations.

Supplementary Table S4 Univariate binary logistic regression analysis for recurrence.

Supplementary Table S5 Multivariate binary logistic regression analysis for recurrence.

Supplementary Table S6 Comparison of microbial findings in this study with relevant results from previous studies.

## References

[B1] Allen N. L. Leeth R. R. Finan K. R. Tishler D. S. Vickers S. M. Wilcox C. M.. (2006). Outcomes of cholecystectomy after endoscopic sphincterotomy for choledocholithiasis. J. Gastrointest. Surg. 10, 292–296. 10.1016/j.gassur.2005.05.013 16455464

[B2] American Association for Cancer Research (2021). *Enterococcus faecalis* colonization in the gut promotes liver carcinogenesis. Cancer Discov. 11:2955. 10.1158/2159-8290.CD-RW2021-143 34625428

[B3] Armbruster C. E. Mobley H. L. (2012). Merging mythology and morphology: the multifaceted lifestyle of *Proteus mirabilis*. Nat. Rev. Microbiol. 10, 743–754. 10.1038/nrmicro2890 23042564 PMC3621030

[B4] Awoniyi M. Wang J. Ngo B. Meadows V. Tam J. Viswanathan A.. (2023). Protective and aggressive bacterial subsets and metabolites modify hepatobiliary inflammation and fibrosis in a murine model of PSC. Gut. 72, 671–685. 10.1136/gutjnl-2021-326500 35705368 PMC9751228

[B5] Ballal M. Jyothi K. N. Antony B. Arun C. Prabhu T. Shivananda P. G. (2001). Bacteriological spectrum of cholecystitis and its antibiogram. Indian J. Med. Microbiol. 19, 212–214. 17664836

[B6] Barahona Ponce C. Scherer D. Brinster R. Boekstegers F. Marcelain K. Gárate-Calderón V.. (2021). Gallstones, body mass index, c-reactive protein, and gallbladder cancer: mendelian randomization analysis of chilean and european genotype data. Hepatology 73, 1783–1796. 10.1002/hep.31537 32893372

[B7] Begley M. Gahan C. G. Hill C. (2005). The interaction between bacteria and bile. FEMS Microbiol. Rev. 29, 625–651. 10.1016/j.femsre.2004.09.003 16102595

[B8] Binh N. T. Ly N. L. Hien P. N. Linh L. T. Lenh B. V. Duc N. M. (2022). Percutaneous transhepatic cholecystolithotomy by holmium laser for non-high-risk patients with symptomatic gallbladder stones. Medical Arch.. 76, 29–33. 10.5455/medarh.2022.76.29-33 35422566 PMC8976887

[B9] Blesl A. Stadlbauer V. (2021). The gut-liver axis in cholestatic liver diseases. Nutrients. 13:1018. 10.3390/nu13031018 33801133 PMC8004151

[B10] Boeder A. M. Spiller F. Carlstrom M. Izídio G. S. (2024). *Enterococcus faecalis*: implications for host health. World J. Microbiol. Biotechnol. 40, 190. 10.1007/s11274-024-04007-w 38702495

[B11] Boerma D. Rauws E. A. Keulemans Y. C. Janssen I. M. Bolwerk C. J. Timmer R.. (2002). Wait-and-see policy or laparoscopic cholecystectomy after endoscopic sphincterotomy for bile-duct stones: a randomised trial. Lancet. 360, 761–765. 10.1016/S0140-6736(02)09896-3 12241833

[B12] Britt N. S. Potter E. M. (2016). Clinical epidemiology of vancomycin-resistant Enterococcus gallinarum and *Enterococcus casseliflavus* bloodstream infections. J. Global Antimicrob. Resist. 5, 57–61. 10.1016/j.jgar.2015.12.002 27274980 PMC4889110

[B13] Brook I. (1989). Aerobic and anaerobic microbiology of biliary tract disease. J. Clin. Microbiol. 27, 2373–2375. 10.1128/jcm.27.10.2373-2375.1989 2584384 PMC267027

[B14] Cheon Y. K. Lehman G. A. (2006). Identification of risk factors for stone recurrence after endoscopic treatment of bile duct stones. Eur. J. Gastroenterol. Hepatol. 18, 461–464. 10.1097/00042737-200605000-00001 16607138

[B15] Choe J. W. Lee J. M. Hyun J. J. Lee H. S. (2021). Analysis on microbial profiles & components of bile in patients with recurrent CBD stones after endoscopic CBD stone removal: a preliminary study. J. Clin. Med. 10:3303. 10.3390/jcm10153303 34362087 PMC8347313

[B16] Citterio B. Francesca B. (2015). Aeromonas hydrophila virulence. Virulence 6, 417–418. 10.1080/21505594.2015.1058479 26055576 PMC4601520

[B17] Cobo T. Vives I. Rodríguez-Trujillo A. Murillo C. Ángeles M. A. Bosch J.. (2017). Impact of microbial invasion of amniotic cavity and the type of microorganisms on short-term neonatal outcome in women with preterm labor and intact membranes. Acta Obstet. Gynecol. Scand. 96, 570–579. 10.1111/aogs.13095 28094842

[B18] David M. Pouncey A. L. Kerwat R. Habal S. (2021). Klebsiella pneumoniae liver abscess with endophthalmitis in a diabetic man with gallstones. BMJ Case Rep. 14:239835. 10.1136/bcr-2020-239835 33637502 PMC7919547

[B19] De Simone B. Abu-Zidan F. M. Chouillard E. Di Saverio S. Sartelli M. Podda M.. (2022). The ChoCO-W prospective observational global study: Does COVID-19 increase gangrenous cholecystitis? World J. Emerg. Surg. 17:61. 10.1186/s13017-022-00466-4 36527038 PMC9755784

[B20] Deng L. Zhen W. Wang J. Lin D. (2023). Bile carriage of optrA-positive *Enterococcus faecium* in a patient with choledocholith. Microbiol. Spect. 11:e0285222. 10.1128/spectrum.02852-22 36976027 PMC10101025

[B21] Englesbe M. J. Dawes L. G. (2005). Resistant pathogens in biliary obstruction: importance of cultures to guide antibiotic therapy. HPB 7, 144–148. 10.1080/13651820510028792 18333179 PMC2023940

[B22] Feng R. Zhang T. Kayani M. U. R. Wang Z. Shen Y. Su K. L.. (2022). Patients with primary and secondary bile duct stones harbor distinct biliary microbial composition and metabolic potential. Front. Cell Infec. Microbiol. 12:881489. 10.3389/fcimb.2022.881489 35548466 PMC9082501

[B23] Gomes P. Fernando N. Weerasekara D. Velathanthiri V. Rizny M. Weerasekera M.. (2009). Aerobic bacteria associated with symptomatic gallstone disease and their antimicrobial susceptibility. Galle Med. J. 11:1110. 10.4038/gmj.v11i1.1110

[B24] Granel-Villach L. Gil-Fortuño M. Fortea-Sanchis C. Gamón-Giner R. L. Martínez-Ramos D. Escrig-Sos V. J. (2020). Factors that influence bile fluid microbiology in cholecystectomized patients. Revista de gastroenterologia de Mexico (English). 85, 257–263. 10.1016/j.rgmx.2019.07.006 32019715

[B25] Grigor'eva I. N. Romanova T. I. (2020). Gallstone disease and microbiome. Microorganisms 8:835. 10.3390/microorganisms8060835 32498344 PMC7356158

[B26] Große K. Ohm D. Würstle S. Brozat J. F. Schmid R. M. Trautwein C.. (2021). Clinical characteristics and outcome of patients with enterococcal liver abscess. Sci. Rep. 11, 22265. 10.1038/s41598-021-01620-9 34782684 PMC8593075

[B27] Helaly G. F. El-Ghazzawi E. F. Kazem A. H. Dowidar N. L. Anwar M. M. Attia N. M. (2014). Detection of Helicobacter pylori infection in Egyptian patients with chronic calcular cholecystitis. Br. J. Biomed. Sci. 71, 13–18. 10.1080/09674845.2014.11669957 24693570

[B28] Hirata B. H. N. Sasagawa S. Navarini A. Mateus H. C. Pacheco Junior A. M. Salles M. J. C. (2023). Comparison of bacterial profile of gallbladder with gallstones from patients undergoing cholecystectomy due to complicated and uncomplicated cholelithiasis: changes in the epidemiological scenario. Rev. Col. Bras. Cir. 50, e20233474. 10.1590/0100-6991e-20233474 37162041 PMC10508671

[B29] Hoogerwerf W. A. Soloway R. D. (1999). Gallstones. Curr. Opin. Gastroenterol. 15, 442–447. 10.1097/00001574-199909000-00012 17023987

[B30] Hooton T. M. Roberts P. L. Cox M. E. Stapleton A. E. (2013). Voided midstream urine culture and acute cystitis in premenopausal women. N. Engl. J. Med. 369, 1883–1891. 10.1056/NEJMoa1302186 24224622 PMC4041367

[B31] Hu H. Shao W. Liu Q. Liu N. Wang Q. Xu J.. (2022). Gut microbiota promotes cholesterol gallstone formation by modulating bile acid composition and biliary cholesterol secretion. Nat. Commun. 13, 252. 10.1038/s41467-021-27758-8 35017486 PMC8752841

[B32] Idowu B. M. Onigbinde S. O. Ebie I. U. Adeyemi M. T. (2019). Gallbladder diseases in pregnancy: sonographic findings in an indigenous African population. J. Ultrasonography. 19, 269–275. 10.15557/JoU.2019.0040 32021708 PMC6988454

[B33] Iida N. Mizukoshi E. Yamashita T. Yutani M. Seishima J. Wang Z.. (2021). Chronic liver disease enables gut *Enterococcus faecalis* colonization to promote liver carcinogenesis. Nature cancer. 2, 1039–1054. 10.1038/s43018-021-00251-3 35121877

[B34] Janjua T. K. Siddique S. Ibrahim M. F. Khurshaidi M. N. (2024). Aeromonas hydrophila induced necrotizing fasciitis following laparoscopic cholecystectomy. JPMA. 74, 576–579. 10.47391/JPMA.9344 38591302

[B35] Joo H. S. Otto M. (2012). Molecular basis of in vivo biofilm formation by bacterial pathogens. Chem. Biol. 19, 1503–1513. 10.1016/j.chembiol.2012.10.022 23261595 PMC3530155

[B36] Konstantakis C. Triantos C. Theopistos V. Theocharis G. Maroulis I. Diamantopoulou G.. (2017). Recurrence of choledocholithiasis following endoscopic bile duct clearance: Long term results and factors associated with recurrent bile duct stones. World J. Gastrointest. Endosc. 9, 26–33. 10.4253/wjge.v9.i1.26 28101305 PMC5215116

[B37] Kose S. H. Grice K. Orsi W. D. Ballal M. Coolen M. J. L. (2018). Metagenomics of pigmented and cholesterol gallstones: the putative role of bacteria. Sci. Rep. 8, 11218. 10.1038/s41598-018-29571-8 30046045 PMC6060111

[B38] Kosowski K. Karczewska E. Kasprowicz A. Andziak J. Heczko P. B. (1987). Bacteria in bile of patients with bile duct inflammation. Eur. J. Clin. Microbiol. 6, 575–578. 10.1007/BF02014251 3436318

[B39] Kwong T. N. Y. Wang X. Nakatsu G. Chow T. C. Tipoe T. Dai R. Z. W.. (2018). Association between bacteremia from specific microbes and subsequent diagnosis of colorectal cancer. Gastroenterology. 155, 383–390.e8. 10.1053/j.gastro.2018.04.028 29729257

[B40] Lazcano-Ponce E. C. Miquel J. F. Muñoz N. Herrero R. Ferrecio C. Wistuba I. I.. (2001). Epidemiology and molecular pathology of gallbladder cancer. CA. Cancer J. Clin. 51, 349–364. 10.3322/canjclin.51.6.349 11760569

[B41] Lee J. Jeong H. J. Kim H. Park J.-S. (2023). The role of the bile microbiome in common bile duct stone development. Biomedicines 11:2124. 10.3390/biomedicines11082124 37626621 PMC10452286

[B42] Lévay B. Szab,ó G Szijártó A Gamal E. M. (2013). The frequency of bacteria in human gallstones. Magy. Seb. 66, 353–356. 10.1556/maseb.66.2013.6.8 24333981

[B43] Liang T. Su W. Zhang Q. Li G. Gao S. Lou J.. (2016). Roles of sphincter of oddi laxity in bile duct microenvironment in patients with cholangiolithiasis: from the perspective of the microbiome and metabolome. J. Am. Coll. Surg. 222, 269-280.e10. 10.1016/j.jamcollsurg.2015.12.009 26922601

[B44] Ljungh A. Wadström T. (2002). The role of microorganisms in biliary tract disease. Curr. Gastroenterol. Rep. 4, 167–171. 10.1007/s11894-002-0055-6 11900683

[B45] Lv F. Zhang S. Ji M. Wang Y. Li P. Han W. (2016). Single-stage management with combined tri-endoscopic approach for concomitant cholecystolithiasis and choledocholithiasis. Surg. Endosc. 30, 5615–5620. 10.1007/s00464-016-4918-6 27126621 PMC5112286

[B46] Man S. Gao Y. Lv J. Tong M. Yin J. Wang B.. (2022). Metabolically healthy obesity was significantly associated with increased risk of gallstones. Eur. J. Endocrinol. 186, 275–283. 10.1530/EJE-21-0802 34889778

[B47] Maurer K. J. Ihrig M. M. Rogers A. B. Ng V. Bouchard G. Leonard M. R.. (2005). Identification of cholelithogenic enterohepatic helicobacter species and their role in murine cholesterol gallstone formation. Gastroenterology 128, 1023–1033. 10.1053/j.gastro.2005.01.008 15825083

[B48] Meng C. Liu K. (2023). Higher levels of systemic immune-inflammatory index are associated with the prevalence of gallstones in people under 50 years of age in the United States: a cross-sectional analysis based on NHANES. Front. Med. 10, 1320735. 10.3389/fmed.2023.1320735 38283040 PMC10811245

[B49] Molinero N. Ruiz L. Milani C. Gutiérrez-Díaz I. Sánchez B. Mangifesta M.. (2019). The human gallbladder microbiome is related to the physiological state and the biliary metabolic profile. Microbiome. 7, 100. 10.1186/s40168-019-0712-8 31272480 PMC6610825

[B50] Muratori R. Mandolesi D. Pierantoni C. Festi D. Colecchia A. Mazzella G.. (2017). Ductal stones recurrence after extracorporeal shock wave lithotripsy for difficult common bile duct stones: Predictive factors. Dig. Liver Dis. 49, 1128–1132. 10.1016/j.dld.2017.05.010 28625406

[B51] Narciso-Schiavon J. L. Borgonovo A. Marques P. C. Tonon D. Bansho E. T. Maggi D. C.. (2015). *Enterococcus casseliflavus* and *Enterococcus gallinarum* as causative agents of spontaneous bacterial peritonitis. Ann. Hepatol. 14, 270–272. 10.1016/S1665-2681(19)30791-4 25671838

[B52] Nardone G. Ferber I. A. Miller L. J. (1995). The integrity of the cholecystokinin receptor gene in gallbladder disease and obesity. Hepatology 22, 1751–1753. 10.1002/hep.1840220621 7489984

[B53] Neri V. Margiotta M. de Francesco V. Ambrosi A. Valle N. D. Fersini A.. (2005). DNA sequences and proteic antigens of *H. pylori* in cholecystic bile and tissue of patients with gallstones. Aliment. Pharmacol. Ther. 22, 715–720. 10.1111/j.1365-2036.2005.02644.x 16197492

[B54] Norsworthy A. N. Pearson M. M. (2017). From catheter to kidney stone: the uropathogenic lifestyle of proteus mirabilis. Trends Microbiol. 25, 304–315. 10.1016/j.tim.2016.11.015 28017513 PMC5365347

[B55] Okumura K. Shoji F. Yoshida M. Mizuta A. Makino I. Higashi H. (2011). Severe sepsis caused by *Aeromonas hydrophila* in a patient using tocilizumab: a case report. J. Med. Case Rep. 5, 499. 10.1186/1752-1947-5-499 21970314 PMC3214171

[B56] Pagani M. A. J. Dolfini P. M. Trazzi B. F. M. Dolfini M. I. M. da Silva W. S. Chagas E. F. B.. (2023). Incidence of bacteriobilia and the correlation with antibioticoprophylaxis in low-risk patients submitted to elective videolaparoscopic cholecystectomy: a randomized clinical trial. Antibiotics. 12:1480. 10.3390/antibiotics12101480 37887181 PMC10604456

[B57] Park W. Park J. (2024). A comparative investigation of the bile microbiome in patients with choledocholithiasis and cholecystolithiasis through metagenomic analysis. Int. J. Mol. Sci. 25:3297. 10.3390/ijms25063297 38542271 PMC10970684

[B58] Peng Y. Yang Y. Liu Y. Nie Y. Xu P. Xia B.. (2015). Cholesterol gallstones and bile host diverse bacterial communities with potential to promote the formation of gallstones. Microb. Pathog. 83–84, 57–63. 10.1016/j.micpath.2015.05.002 25959528

[B59] Ploszaj T. Brauncajs M. Traczyk-Borszynska M. Matyjas T. Pomorski L. Wasiak T.. (2021). The value of bacterial metagenomic analysis in post-surgical examination of gallstones. Arch. Microbiol. 203, 6323–6328. 10.1007/s00203-021-02580-4 34562145 PMC8590668

[B60] Portincasa P. Di Ciaula A. Bonfrate L. Stella A. Garruti G. Lamont J. T. (2023). Metabolic dysfunction-associated gallstone disease: expecting more from critical care manifestations. Intern. Emerg. Med. 18, 1897–1918. 10.1007/s11739-023-03355-z 37455265 PMC10543156

[B61] Ryu S. Chang Y. Yun K. E. Jung H. S. Shin J. H. Shin H. (2016). Gallstones and the risk of gallbladder cancer mortality: a cohort study. Am. J. Gastroenterol. 111, 1476–1487. 10.1038/ajg.2016.345 27575712

[B62] Sacks D. Baxter B. Campbell B. C. V. Carpenter J. S. Cognard C. Dippel D.. (2018). Multisociety consensus quality improvement revised consensus statement for endovascular therapy of acute ischemic stroke. Int. J. Stroke. 13, 612–632. 10.1016/j.jvir.2017.11.026 29786478

[B63] Sattar I. Aziz A. Rasul S. Mehmood Z. Khan A. (2007). Frequency of infection in cholelithiasis. J. College of Physi. Surg.–Pakistan: JCPSP. 17, 48–50. 17204221

[B64] Serraino C. Elia C. Bracco C. Rinaldi G. Pomero F. Silvestri A.. (2018). Characteristics and management of pyogenic liver abscess: a European experience. Medicine 97:e0628. 10.1097/MD.0000000000010628 29742700 PMC5959441

[B65] Shabanzadeh D. M. Holmboe S. A. Sørensen L. T. Linneberg A. Andersson A. M. Jørgensen T. (2017). Are incident gallstones associated to sex-dependent changes with age? a cohort study. Andrology. 5, 931–938. 10.1111/andr.12391 28704597

[B66] Shabanzadeh D. M. Sørensen L. T. Jørgensen T. (2016). Abdominal symptoms and incident gallstones in a population unaware of gallstone status. Can. J. Gastroenterol. Hepatol. 2016:9730687. 10.1155/2016/9730687 27504440 PMC4967696

[B67] Shabanzadeh D. M. Sørensen L. T. Jørgensen T. (2019). Determinants for symptomatic gallstone disease readmissions - results from a cohort with screen-detected gallstone disease. J. Visc. Surg. 156, 387–396. 10.1016/j.jviscsurg.2019.02.005 30824211

[B68] Shen H. Ye F. Xie L. Yang J. Li Z. Xu P.. (2015). Metagenomic sequencing of bile from gallstone patients to identify different microbial community patterns and novel biliary bacteria. Sci. Rep. 5, 17450. 10.1038/srep17450 26625708 PMC4667190

[B69] Shen H. Zhu J. Ye F. Xu D. Fang L. Yang J.. (2020). Biliary microbial structure of gallstone patients with a history of endoscopic sphincterotomy surgery. Front. Cell. Infect. Microbiol. 10:594778. 10.3389/fcimb.2020.594778 33585269 PMC7873689

[B70] Song S. T. Shi J. Wang X. H. Guo Y. B. Hu P. F. Zhu F.. (2020). Prevalence and risk factors for gallstone disease: a population-based cross-sectional study. J. Dig. Dis. 21, 237–245. 10.1111/1751-2980.12857 32166900

[B71] Song Y. Ma Y. Xie F. C. Jin C. Yang X. B. Yang X.. (2022). Age, gender, geographic and clinical differences for gallstones in China: a nationwide study. Ann. Transl. Med. 10, 735. 10.21037/atm-21-6186 35957733 PMC9358507

[B72] Stekhoven D. J. Bühlmann P. (2012). MissForest–non-parametric missing value imputation for mixed-type data. Bioinformatics 28, 112–118. 10.1093/bioinformatics/btr597 22039212

[B73] Stewart L. Grifiss J. M. Jarvis G. A. Way L. W. (2006). Biliary bacterial factors determine the path of gallstone formation. Am. J. Surg. 192, 598–603. 10.1016/j.amjsurg.2006.08.001 17071191

[B74] Stewart L. Oesterle A. L. Erdan I. Griffiss J. M. Way L. W. (2002). Pathogenesis of pigment gallstones in Western societies: the central role of bacteria. J. Gastrointest. Surg. 6, 891–903. 10.1016/S1091-255X(02)00035-5 12504229

[B75] Sun H. Warren J. Yip J. Ji Y. Hao S. Han W.. (2022). Factors influencing gallstone formation: a review of the literature. Biomolecules 12:550. 10.3390/biom12040550 35454138 PMC9026518

[B76] Swidsinski A. Lee S. P. (2001). The role of bacteria in gallstone pathogenesis. Front. Biosci. 6, E93–103. 10.2741/A699 11578976

[B77] Tajeddin E. Sherafat S. J. Majidi M. R. Alebouyeh M. Alizadeh A. H. Zali M. R. (2016). Association of diverse bacterial communities in human bile samples with biliary tract disorders: a survey using culture and polymerase chain reaction-denaturing gradient gel electrophoresis methods. Eur. J. Clin. Microbiol. Infect. Dis. 35, 1331–1339. 10.1007/s10096-016-2669-x 27193890

[B78] Tan W. Chen R. Song J. He D. Wu J. Chen X.. (2022). Microbiota analysis with next-generation 16S rDNA gene sequencing in recurrent common bile duct stones. Ann. Transl. Med. 10:576. 10.21037/atm-22-2247 35722401 PMC9201157

[B79] Tanaka H. Imasato M. Yamazaki Y. Matsumoto K. Kunimoto K. Delpierre J.. (2018). Claudin-3 regulates bile canalicular paracellular barrier and cholesterol gallstone core formation in mice. J. Hepatol. 69, 1308–1316. 10.1016/j.jhep.2018.08.025 30213590

[B80] Tsuchiya Y. Loza E. Villa-Gomez G. Trujillo C. C. Baez S. Asai T.. (2018). Metagenomics of microbial communities in gallbladder bile from patients with gallbladder cancer or cholelithiasis. Asian Pac. J. Cancer Prev. 19, 961–967. 29693356 10.22034/APJCP.2018.19.4.961 PMC6031792

[B81] Vitetta L. Best S. P. Sali A. (2000). Single and multiple cholesterol gallstones and the influence of bacteria. Med. Hypotheses. 55, 502–506. 10.1054/mehy.2000.1101 11090298

[B82] von Schönfels W. Buch S. Wölk M. Aselmann H. Egberts J. H. Schreiber S.. (2013). Recurrence of gallstones after cholecystectomy is associated with ABCG5/8 genotype. J. Gastroenterol. 48, 391–396. 10.1007/s00535-012-0639-3 22869156

[B83] Wang H. H. Portincasa P. Afdhal N. H. Wang D. Q. (2010). Lith genes and genetic analysis of cholesterol gallstone formation. Gastroenterol. Clin. North Am. 39, 185–207, vii-viii. 10.1016/j.gtc.2010.02.007 20478482

[B84] Wang Y. Qi M. Qin C. Hong J. (2018). Role of the biliary microbiome in gallstone disease. Expert Rev. Gastroenterol. Hepatol. 12, 1193–1205. 10.1080/17474124.2018.1533812 30791792

[B85] Wu T. Zhang Z. Liu B. Hou D. Liang Y. Zhang J.. (2013). Gut microbiota dysbiosis and bacterial community assembly associated with cholesterol gallstones in large-scale study. BMC Genomics. 14, 669. 10.1186/1471-2164-14-669 24083370 PMC3851472

[B86] Xie Y. Q. Zhang J. Z. Zhang H. Peng L. Zhou S. Li L. Z.. (2016). Pregnane receptor gene polymorphisms, pathogenic bacteria distribution and drug sensitivity, and TCM syndrome differentiation in patients with cholelithiasis. Asian Pac. J. Trop. Med. 9, 307–312. 10.1016/j.apjtm.2016.03.001 27086146

[B87] Yang X. T. Wang J. Jiang Y. H. Zhang L. Du L. Li J.. (2023). Insight into the mechanism of gallstone disease by proteomic and metaproteomic characterization of human bile. Front. Microbiol. 14:1276951. 10.3389/fmicb.2023.1276951 38111640 PMC10726133

[B88] Ye C. Zhou W. Zhang H. Miao L. Lv G. (2020). Alterations of the Bile Microbiome in Recurrent Common Bile Duct Stone. Biomed Res. Int. 2020:4637560. 10.1155/2020/4637560 33062679 PMC7542479

[B89] Yoo E. H. Lee S. Y. (2009). The prevalence and risk factors for gallstone disease. Clin. Chem. Lab. Med. 47, 795–807. 10.1515/CCLM.2009.194 19499973

[B90] Yoshino Y. (2023). Enterococcus casseliflavus Infection: A Review of Clinical Features and Treatment. Infect. Drug Resist. 16, 363–368. 10.2147/IDR.S398739 36714353 PMC9879772

[B91] Zhang Y. Sun L. Wang X. Chen Z. (2022). The association between hypertension and the risk of gallstone disease: a cross-sectional study. BMC Gastroenterol. 22:138. 10.1186/s12876-022-02149-5 35346065 PMC8961935

[B92] Zhu Q. Xing Y. Fu Y. Chen X. Guan L. Liao F.. (2023). Causal association between metabolic syndrome and cholelithiasis: a Mendelian randomization study. Front Endocrinol. 14:1180903. 10.3389/fendo.2023.1180903 37361524 PMC10288183

