# Supplementary figures and images for: Microbial characteristics of bile in gallstone patients: a comprehensive analysis of 9,939 cases
Source: Front Microbiol. 2024 Dec 19;15:1481112. doi: 10.3389/fmicb.2024.1481112 (PMC11693992; doi:10.3389/fmicb.2024.1481112)

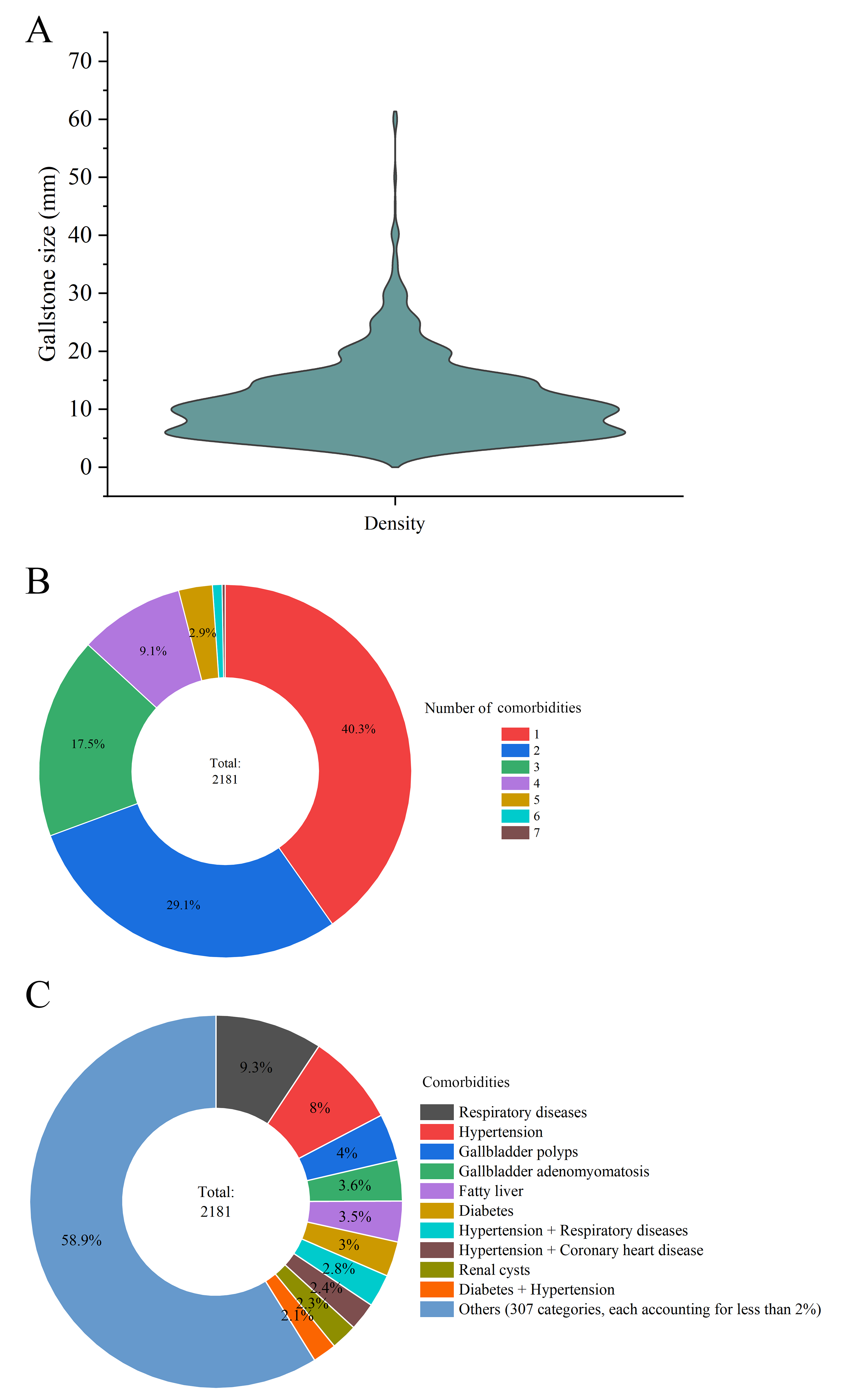

Supplement: Supplementary Figure S1 — Stone size and comorbidity information. (A) Distribution of stone sizes. This figure clearly shows the range and concentration trend of stone sizes. The horizontal axis represents patient density, and the vertical axis represents stone size. The wider the section, the more patients fall within that stone size range. (B) Number and proportion of comorbidities. Each patient belongs to only one category. For example, the red section indicates that 40.3% of patients have only one comorbidity, while the blue section shows that 29.1% of patients have two comorbidities. Only a very small portion of patients have six or seven comorbidities simultaneously. (C) Categories and proportions of comorbidity combinations. Each patient belongs to only one category. Each patient belongs to only one category, meaning each patient is counted under one comorbidity combination. This classification helps identify the most common comorbidity combinations and their distribution. For example, gallstone patients with only respiratory system diseases represent the largest group (9.31%), followed by those with only hypertension (8.02%). [file Image_1.tif]
