# Supplementary material for: Microbial characteristics of bile in gallstone patients: a comprehensive analysis of 9,939 cases
Source: Front Microbiol. 2024 Dec 19;15:1481112. doi: 10.3389/fmicb.2024.1481112 (PMC11693992; doi:10.3389/fmicb.2024.1481112)
Supplement: Supplementary Table S4 — Univariate binary logistic regression analysis for recurrence. [file Table_3.docx]

Table S4. Univariate binary logistic regression analysis for recurrence.

| Variable | OR | 95%CI | P value |
| --- | --- | --- | --- |
| Stone size | 1.08 | 1.05-1.10 | ＜0.001 |
| Diabetes | 2.06 | 1.38 -3.09 | ＜0.001 |
| Renal cyst | 2.357 | 1.453-3.825 | ＜0.001 |
| Venous thrombosis | 3.23 | 1.99-5.25 | ＜0.001 |
| Coronary heart disease | 1.89 | 1.26-2.82 | 0.002 |
| Cirrhosis of the liver | 17.41 | 6.85-44.21 | ＜0.001 |
| Malignant tumor | 3.15 | 1.66-5.99 | ＜0.001 |
| Respiratory diseases | 2.70 | 1.84-3.97 | ＜0.001 |
| Extrahepatic bile duct stones | 1.95 | 1.33-2.88 | ＜0.001 |
| Cholecystolithiasis | 0.42 | 0.28-0.64 | ＜0.001 |
| Hepatolithiasis bile duct stones | 2.81 | 1.32-5.97 | 0.007 |
| Number of species | 2.23 | 1.49-1.85 | ＜0.001 |

OR represents the odds ratio, CI indicates the confidence interval, and P value signifies statistical significance.

Table S5. Multivariate Binary logistic regression analysis for recurrence.

| Variable | OR | 95%CI | P value |
| --- | --- | --- | --- |
| Age | 0.97 | 0.95-0.99 | 0.001 |
| Stone size | 1.06 | 1.04-1.08 | ＜0.001 |
| Diabetes | 2.45 | 1.51-3.96 | ＜0.001 |
| Venous thrombosis | 2.94 | 1.56-5.52 | 0.001 |
| Coronary heart disease | 1.93 | 1.14-3.26 | 0.014 |
| Cirrhosis of the liver | 5.71 | 1.99-16.34 | 0.001 |
| Malignant tumor | 3.26 | 1.51-7.07 | 0.003 |
| Number of species | 2.89 | 1.77-4.71 | ＜0.001 |

OR represents the odds ratio, CI indicates the confidence interval, and P value signifies statistical significance.

Table S6. Comparison of microbial findings in this study with relevant results from previous studies.

| **Microbe** | **Results in this study** | **Relevant results from other studies** |
| --- | --- | --- |
| *E. coli* | - The dominant species; - The highest positive detection frequency in the ≥75-year-old group among the four different age groups; - The EBD group had a significantly higher positive detection frequency compared to the cholecystolithiasis group among the different gallstone types; - Significant difference before and after recurrence, appearing in more patients after recurrence. | - The dominant species [1-6]; - One of the causative agents of gallstone formation [2, 7]; - Culture results indicate that biofilm-forming bacteria (*Pseudomonas aeruginosa*, *E. coli*, *K. pneumoniae*, *Enterococcus* spp., and *Acinetobacter* spp.) coexist in various combinations and are the primary bacteria associated with gallstone [2, 6]. |
| *Klebsiella pneumoniae* | - The dominant species; - The highest positive detection frequency in the ≥75-year-old group among the four different age groups; - The EBD group had a significantly higher positive detection frequency compared to the cholecystolithiasis group among the different gallstone types; - Significant difference before and after recurrence, appearing in more patients after recurrence. | - The dominant species [1-6]; - Common in East Asian populations, usually associated with diabetes and gallstones [8, 9]; - Culture results indicate that biofilm-forming bacteria (*P. aeruginosa*, *E. coli*, *K. pneumoniae*, *Enterococcus* spp., and *Acinetobacter* spp.) coexist in various combinations and are the primary bacteria associated with gallstone [2, 6]. |
| *Enterococcus faecalis* | - The dominant species; - The highest positive detection frequency in the ≥75-year-old group among the four different age groups; - The EBD group had a significantly higher positive detection frequency compared to the cholecystolithiasis group among the different gallstone types. | - The dominant species [1-6]; - Associated with human infections and primarily affects immunocompromised individuals [10]; - Abundant in patients with chronic hepatobiliary diseases [11-14]; - Can induce hepatic tumorigenesis [11, 15]; - Culture results indicate that biofilm-forming bacteria (*P. aeruginosa*, *E. coli*, *K. pneumoniae*, *Enterococcus* spp., and *Acinetobacter* spp.) coexist in various combinations and are the primary bacteria associated with gallstone [2, 6]. |
| *Pseudomonas aeruginosa* | - The HBD group had a significantly higher positive detection frequency compared to the cholecystolithiasis and EBD groups among the different gallstone types; - Significant difference before and after recurrence, appearing in more patients after recurrence. | - 30% of cholesterol gallstones can be cultured in strains secreting β-glucuronidase and phospholipase A2, and Pseudomonas aeruginosa has the highest β-glucuronidase activity, which may be a major factor in the formation of cholesterol gallstones [16]; - Culture results indicate that biofilm-forming bacteria (*P. aeruginosa*, *E. coli*, *K. pneumoniae*, *Enterococcus* spp., and *Acinetobacter* spp.) coexist in various combinations and are the primary bacteria associated with gallstone [2, 6]. |
| *Aeromonas hydrophila* | Significant difference before and after recurrence, appearing in more patients after recurrence. | - Can cause several serious infections such as gastroenteritis, skin infections, peritonitis, bacteremia, meningitis, and necrotizing fasciitis [17]; - A 72-year-old female patient died of necrotizing fasciitis caused by Aeromonas hydrophila after undergoing laparoscopic cholecystectomy for gallstones with hypertension [18]; - A 72-year-old patient with gallstones and rheumatoid arthritis using the immunosuppressive drug tocilizumab developed Aeromonas hydrophila sepsis and acute septic cholangitis [19]. |
| *Enterococcus casseliflavus* | Significant difference before and after recurrence, appearing in more patients after recurrence. | Has a high affinity for the biliary system and is prone to cause infections of the biliary tract and liver [20]. |
| *Enterococcus faecium* | Significant difference before and after recurrence, appearing in more patients after recurrence. | - Resistant to a variety of antibiotics, including vancomycin and oxazolidinone antibiotics[10]; - In 2021, an *E. faecium* strain carrying the oxazolidinone resistance gene was found in a bile sample from a patient with choledocholithiasis in Shenzhen, China, demonstrating its resistance and complexity in the biliary system [21]. |
| *Proteus mirabilis* | Significant difference before and after recurrence, appearing in more patients after recurrence. | Known for its ability to produce urease, an enzyme that breaks down urea to produce ammonia, alkalizing the local environment and predisposing to stone formation [22, 23]. |
| *Microbial diversity* | Significantly lower in the recurrence group compared to the non-recurrence group. | - The microbial richness was significantly lower in the recurrence group than in the non-recurrence group, and microbial homogeneity was also reduced, with differences in the overall microbial communities between the two groups [24]; - The diversity of the biliary microbial community was significantly lower in patients with recurrent choledocholithiasis [25]. |

# References

1. Lévay, B., et al., *[The frequency of bacteria in human gallstones].* Magy Seb, 2013. **66**(6): p. 353-6.

2. Tajeddin, E., et al., *Association of diverse bacterial communities in human bile samples with biliary tract disorders: a survey using culture and polymerase chain reaction-denaturing gradient gel electrophoresis methods.* Eur J Clin Microbiol Infect Dis, 2016. **35**(8): p. 1331-9.

3. Sattar, I., et al., *Frequency of infection in cholelithiasis.* J Coll Physicians Surg Pak, 2007. **17**(1): p. 48-50.

4. Gomes, P., et al., *Aerobic bacteria associated with symptomatic gallstone disease and their antimicrobial susceptibility.* Galle Medical Journal, 2009. **11**.

5. Ballal, M., et al., *Bacteriological spectrum of cholecystitis and its antibiogram.* Indian J Med Microbiol, 2001. **19**(4): p. 212-4.

6. Pagani, M.A.J., et al., *Incidence of Bacteriobilia and the Correlation with Antibioticoprophylaxis in Low-Risk Patients Submitted to Elective Videolaparoscopic Cholecystectomy: A Randomized Clinical Trial.* Antibiotics (Basel), 2023. **12**(10).

7. Blesl, A. and V. Stadlbauer, *The Gut-Liver Axis in Cholestatic Liver Diseases.* Nutrients, 2021. **13**(3).

8. Serraino, C., et al., *Characteristics and management of pyogenic liver abscess: A European experience.* Medicine (Baltimore), 2018. **97**(19): p. e0628.

9. David, M., et al., *Klebsiella pneumoniae liver abscess with endophthalmitis in a diabetic man with gallstones.* BMJ Case Rep, 2021. **14**(2).

10. Boeder, A.M., et al., *Enterococcus faecalis: implications for host health.* World J Microbiol Biotechnol, 2024. **40**(6): p. 190.

11. *Enterococcus faecalis Colonization in the Gut Promotes Liver Carcinogenesis.* Cancer Discov, 2021. **11**(12): p. 2955.

12. Awoniyi, M., et al., *Protective and aggressive bacterial subsets and metabolites modify hepatobiliary inflammation and fibrosis in a murine model of PSC.* Gut, 2023. **72**(4): p. 671-685.

13. Große, K., et al., *Clinical characteristics and outcome of patients with enterococcal liver abscess.* Sci Rep, 2021. **11**(1): p. 22265.

14. Xie, Y.Q., et al., *Pregnane receptor gene polymorphisms, pathogenic bacteria distribution and drug sensitivity, and TCM syndrome differentiation in patients with cholelithiasis.* Asian Pac J Trop Med, 2016. **9**(4): p. 307-312.

15. Iida, N., et al., *Chronic liver disease enables gut Enterococcus faecalis colonization to promote liver carcinogenesis.* Nat Cancer, 2021. **2**(10): p. 1039-1054.

16. Peng, Y., et al., *Cholesterol gallstones and bile host diverse bacterial communities with potential to promote the formation of gallstones.* Microb Pathog, 2015. **83-84**: p. 57-63.

17. Citterio, B. and B. Francesca, *Aeromonas hydrophila virulence.* Virulence, 2015. **6**(5): p. 417-8.

18. Janjua, T.K., et al., *Aeromonas hydrophila induced necrotizing fasciitis following laparoscopic cholecystectomy.* J Pak Med Assoc, 2024. **74**(3): p. 576-579.

19. Okumura, K., et al., *Severe sepsis caused by Aeromonas hydrophila in a patient using tocilizumab: a case report.* J Med Case Rep, 2011. **5**: p. 499.

20. Yoshino, Y., *Enterococcus casseliflavus Infection: A Review of Clinical Features and Treatment.* Infect Drug Resist, 2023. **16**: p. 363-368.

21. Deng, L., et al., *Bile Carriage of optrA-Positive Enterococcus faecium in a Patient with Choledocholith.* Microbiol Spectr, 2023. **11**(2): p. e0285222.

22. Armbruster, C.E. and H.L. Mobley, *Merging mythology and morphology: the multifaceted lifestyle of Proteus mirabilis.* Nat Rev Microbiol, 2012. **10**(11): p. 743-54.

23. Norsworthy, A.N. and M.M. Pearson, *From Catheter to Kidney Stone: The Uropathogenic Lifestyle of Proteus mirabilis.* Trends Microbiol, 2017. **25**(4): p. 304-315.

24. Choe, J.W., et al., *Analysis on Microbial Profiles & Components of Bile in Patients with Recurrent CBD Stones after Endoscopic CBD Stone Removal: A Preliminary Study.* J Clin Med, 2021. **10**(15).

25. Tan, W., et al., *Microbiota analysis with next-generation 16S rDNA gene sequencing in recurrent common bile duct stones.* Ann Transl Med, 2022. **10**(10): p. 576.
